# Bronchial epithelial cell-derived extracellular vesicles drive inflammasome activation and *NTHi* infection in COPD

**DOI:** 10.3389/fimmu.2025.1713012

**Published:** 2026-01-02

**Authors:** Georgia Bateman, Hong Guo-Parke, Caitlyn Harvey, Aoife Rodgers, Anna Krasnodembskaya, Dermot Linden, Rabindra Tirouvanziam, Lee A. Borthwick, Andrew J. Fisher, Judith Coppinger, Joe Kidney, Clifford C. Taggart

**Affiliations:** 1 Airway Innate Immunity Research (AiiR) Group, Wellcome-Wolfson Institute for Experimental Medicine, School of Medicine, Dentistry and Biomedical Sciences, Queen’s University Belfast, Belfast, United Kingdom; 2 Department of Pediatrics, Emory University School of Medicine, Atlanta, GA, United States; 3 Center for Cystic Fibrosis (CF) and Airways Disease Research, Children’s Healthcare of Atlanta, Atlanta, GA, United States; 4 Newcastle Fibrosis Research Group, Biosciences Institute, Faculty of Medical Sciences, Newcastle University, Newcastle Upon Tyne, United Kingdom; 5 Institute of Transplantation, Newcastle Upon Tyne Hospitals National Health Service (NHS) Foundation Trust, Newcastle Upon Tyne, United Kingdom; 6 Transplantation and Regenerative Medicine, Translational and Clinical Research Institute, Faculty of Medical Sciences, Newcastle University, Newcastle Upon Tyne, United Kingdom; 7 School of Pharmacy and Biomolecular Sciences, St. Stephen’s Green, Dublin, Ireland; 8 Department of Respiratory Medicine, Mater Hospital, Belfast, United Kingdom

**Keywords:** extracellular vesicles, COPD, NLRP3, macrophages, *NTHi*

## Abstract

Extracellular vesicles (EVs) are lipid-membrane bound vesicles that can be beneficial or detrimental depending on the content they carry. As epithelial cells are the first line of defense against harmful particles, this work explored the role of bronchial epithelial cell-derived EVs (CepEVs) in the pathogenesis and progression of chronic obstructive pulmonary disease (COPD). RNA sequencing of macrophages stimulated with CepEVs revealed the upregulation of various inflammasome-related genes, alongside significant IL-1b and IL-18 release, which could be attenuated with caspase-1 or NLRP3 inhibition. The proteome of CepEVs was also assessed, which highlighted a significant reduction in antibacterial proteins compared to healthy EVs (HepEVs). When functionally assessed in *NTHi* infection of THP-1 cells, pre-incubation with HepEVs stimulated *NTHi* clearance and reduced pro-inflammatory cytokine release by macrophages, which was reduced in CepEV-stimulated cells. This study shows for the first time that CepEVs are able to both prime and activate the inflammasome in healthy macrophages, and highlights EV-induced inflammasome inhibition as a potential therapeutic target for the dysregulated inflammation seen in COPD. Alongside the inflammasome, we were also able to show that CepEVs are deficient for multiple antibacterial proteins, and that one or more of these proteins are essential in mounting an immune response against *NTHi* in macrophages. This finding contributes to a potential therapeutic pipeline through the supplementation of the depleted antibacterial proteins in CepEVs, allowing for efficient bacterial clearance and reduced consequential inflammatory burden. CepEV co-incubation resulted in a persistent state of inflammation and infection. Both sets of findings contribute to the overall knowledge of COPD pathogenesis, and highlight epithelial EVs as key players in the propagation of inflammation and susceptibility to infection.

## 1 Introduction

Extracellular vesicles (EVs) are small lipid bound vesicles secreted by all cell types into the extracellular space, and the release of EVs is an evolutionarily conserved process, from bacteria to plants to animals (1, 2). ‘EV’ is an umbrella term encompassing various subtypes of secreted vesicles, which can be distinguished by their biogenesis, release, size, content, and function. The three main subtypes of EVs, termed classical EVs, are exosomes, microvesicles, and apoptotic bodies. As all cells involved in the immune response can secrete EVs, it’s no surprise that EVs play a role in inflammatory processes. It has been shown that EVs can have both pro-inflammatory and anti-inflammatory roles depending on the parent cell type, and the condition the parent cell is in (3).

EVs are involved in the pathology of various inflammatory lung conditions. For example, exosomes originating from eosinophils in asthmatic patients were shown to be produced at higher levels when compared to healthy controls, and promoted further chemotaxis of eosinophils, perpetuating tissue damage and inflammation in an autoregulated cycle (4, 5). In addition, over 140 plasma and sputum EV-miRNAs have been identified that correlate with asthma symptoms and associated tissue damage (6–8).

Chronic obstructive pulmonary disease (COPD) is a global epidemic, the occurrence of which has been steadily increasing over the last five decades, and is associated with a very high socio-economic burden that is expected to increase with the ageing population, increased tobacco and E-cigarette use, and sustained air pollution levels (9, 10). COPD is currently amongst the top ten causes of death worldwide affecting over 12% of the population aged 40 and older, and the World Health Organization (WHO) mortality and disease burden projections predict that COPD will be the third leading cause of death by 2030 (11–13). COPD is characterized by progressive and persistent narrowing of the small airways and/or destruction of the alveolar walls, primarily induced through long-term inhalation of harmful particles, resulting in non-fully reversible airflow limitation (14). These pathological changes result in airway obstruction and alveolar emphysema, the relative contributions of which depend on the individual (15). While present treatments work to slow progression and alleviate symptoms of COPD, there is currently no therapy available to target and correct the underlying mechanisms that result in the airway remodeling driving COPD pathogenesis.

Inflammasomes are multimeric protein complexes that assemble in the cytosol after sensing danger signals such as pathogen- and damage-associated molecular patterns (PAMPs/DAMPs) (16). There are distinct inflammasomes, each of which is activated by various stimuli, as reviewed elsewhere (17). The nucleotide-binding oligomerization domain-like receptor pyrin domain-containing 3 (NLRP3) inflammasome will be the only inflammasome discussed henceforth. The NLRP3 inflammasome consists of a sensor (NLRP3), an adaptor known as the apoptosis-associated speck-like protein containing a caspase recruitment domain (ASC), and an effector (caspase-1) (18). NLRP3, unlike most PAMP/DAMP sensing pattern recognition receptors (PRRs), can be activated by a wide variety of stimuli (19). The assembly and activation of the inflammasome must be tightly regulated due to the potentially feed-forward nature of the process. Inflammasome activation is considered, for the most part, to be a two-step process in macrophages – priming and activation. The role of the priming step is to upregulate the expression of various inflammasome components, namely NLRP3 and pro-IL-1β. The priming step occurs in response to PRRs recognizing PAMPs/DAMPs, such as Toll-like receptors (TLRs) binding to their cognate ligands, referred to as signal one, which, in turn triggers NF-κB-dependent transcription of necessary inflammasome components (20).

Emerging evidence suggests that activation of the NLRP3 inflammasome may be involved in the onset of COPD. While primarily associated with alveolar macrophages, various cell types within the lungs express NLRP3, including type II alveolar epithelial cells, lung fibroblasts, lung-resident mesenchymal stromal cells (MSCs), and endothelial cells (21–24). Elevated levels of IL-1β have been found in the lungs of COPD patients, which are further amplified during exacerbations (25). In addition, it has previously been shown that mice exposed to cigarette smoke (CS) have significantly increased levels of IL-1β, and that IL-1 receptor (IL-1R) knockout mice were protected against CS-induced matrix breakdown and increases in inflammatory cells within bronchoalveolar lavage (BAL) (26). Interestingly, NLRP3 is over-expressed in the lungs of stable COPD patients, which isn’t seen in smokers with normal spirometry, suggesting that NLRP3 expression specifically correlates to airflow obstruction rather than being a direct result of CS exposure (27). Finally, it has been shown that although the mRNA and protein level of caspase-1 was similar between smokers, non-smokers, and COPD patients, levels of active caspase-1 were significantly higher in the sputum of COPD patients (27, 28). In the context of EVs, epithelial EVs have previously been linked to the inflammasome and caspase activation in other disease states. Hypoxia has been shown to induce the release of EVs in the lung epithelial cells that contain high levels of active caspase-3 (29). Alveolar epithelial cells have also been shown to release EVs that induce NLRP3 inflammasome activation in alveolar macrophages in response to bacterial stimuli (30). However, to date, no studies have been done to assess the effect of COPD bronchial epithelial cells on inflammasome activation in macrophages.

As discussed in detail elsewhere (31), human lungs are endowed with a wide range of defense mechanisms to ensure tissue defense and repair even in the face of repeat exposure to inhaled insults. However, in the context of COPD, these defense mechanisms are disrupted, allowing for repeated infections that perpetuate damage to the lungs in a cyclic manner. These repeated infections promote increased inflammation and worsening symptoms, often leading to acute exacerbations of COPD. However, up to 50% of stable COPD patients also carry potentially pathogenic bacteria in their airways (32). It has been long known that there is a positive correlation between the airway bacterial load and inflammation (33). Detection of bacteria in the airway was shown to correlate with levels of pro-inflammatory cytokines such as IL-1β and TNF-α in the sputum (34). Arguably the most common cause of bacterial infection in stable COPD patients is *non-typeable Haemophilus influenzae* (*NTHi*) (35). *NTHi* accounts for up to half of the bacteria isolated from stable COPD patients (36). *NTHi* is a human-restricted, Gram-negative, opportunistic pathogen, despite also having commensal properties (37). Lipooligosaccharide (LOS), a component of the *NTHi* cell wall, alongside P6, an outer membrane protein, are potent immunomodulators of macrophages, inducing IL-8 and TNF-α secretion which in turn promote neutrophil recruitment (38–40). Combined with the dysfunctional phagocytosis displayed by alveolar macrophages in COPD, this secretion promotes an inflammatory, neutrophil-rich, environment with inefficient *NTHi* clearance (41). Interestingly, *NTHi* infection has also been shown to upregulate the NLRP3 inflammasome, further promoting inflammation and acute exacerbations (42). The effect of pre-stimulated epithelial EVs in macrophage bacterial infection have been previously assessed. Lane *et.al*, evaluated the effect of EVs released from the respiratory epithelium after antiviral signaling in monocyte-derived macrophages (MDMs) (43). The survival of *Staphylococcus aureus (S. aureus)* was significantly increased in MDMs exposed to antiviral EVs compared to control epithelial EVs. This study highlights the role of EVs in the epithelium-macrophage infection crosstalk, however this has not been previously assessed in COPD.

In this study, we sought to decipher the role of COPD bronchial epithelial cell-derived EVs (CepEVs) in macrophage function. Firstly, EVs were isolated and characterized. The role of CepEVs in macrophage inflammasome activation was then explored through RNA sequencing analysis and cytokine assessment. Subsequently, use of known inflammasome inhibitors MCC950 and VX-765 was assessed to functionally confirm inflammasome activation. Moreover, our data show that these same EVs affect macrophage susceptibility to *NTHi* infection, and through proteomic analysis have identified key proteins for further study.

## 2 Materials and methods

### 2.1 Primary bronchial epithelial cells

#### 2.1.1 Sample information

COPD primary bronchial epithelial cells (PBECs) were derived from brushings taken from the Epstein-Barr Virus Suppression in Chronic Obstructive Pulmonary Disease (EViSCO) trial (44). The trial was sponsored by Belfast Health and Social Care Trust and received approval from the Office of Research Ethics Committees Northern Ireland (18/NI/0106). The clinical characteristics of COPD patients are detailed in **Supplementary Table 1**. All samples were taken pre-treatment. Age-matched healthy control PBECs were purchased from Lonza or PromoCell (**Supplementary Table 2**).

#### 2.1.2 Air-liquid interface culture

PBECs were seeded in semipermeable Costar^®^ 6.5 mm Transwell^®^ Membrane Inserts (6 mm diameter, 0.4 µm pores, Cat#05001) in 24-well plates (5x10^4^cells/well) and maintained until confluent. Cells were maintained in 200 μL PneumaCult™-Ex Plus Medium (Stemcell™ Technologies, Cat#05040), with 500 μL in the basal chamber. Once 100% confluence was reached, the medium in the apical chamber of the transwell was removed. From this point onward, cells were fed through the basal chamber with PneumaCult™-ALI Medium (Stemcell™ Technologies, Cat#05002). The apical chamber remained exposed to air to promote differentiation into a pseudostratified heterogeneous cell population with occasional washing with PBS required to remove mucus build up. Cells were maintained at air-liquid interface (ALI) until cilia were visibly beating (roughly 3 weeks), with basal media changes every other day. All basal media was collected after this point and frozen at -80°C for later EV isolations.

### 2.2 Macrophage models

#### 2.2.1 THP-1 cell culture

The THP-1 monocytic cell line was maintained in RPMI 1640 (Gibco) supplemented with 10% fetal bovine serum (FBS) and 1% penicillin-streptomycin (Pen-Strep). For differentiation into macrophage-like cells, cells were seeded at 1.25x10^5^ cells/well in 48-well plates with 100 nM/well phorbol-12-myristate 13-acetate (PMA) for 72 hrs. After 72 hrs, PMA-stimulated cells had adhered, PMA-containing media was carefully removed, and cells were incubated in fresh RPMI (10% FBS, 1% Pen-Strep) for 24 hrs before any experimental procedures.

#### 2.2.2 Monocyte-derived macrophages

PBMCs were isolated from whole blood through density gradient centrifugation as previously described (45). Ethical approval for the use of blood was received from the Queen’s University Faculty Research Ethics Committee (reference MHLS 19_17). After isolation, cells were incubated at 37°C, 5% CO_2_ for 1.5 hrs in a 24-well plate to ensure cell adhesion. After the incubation step any non-adherent cells, mainly lymphocytes, were removed by vigorous washing with Hanks balanced salt solution (HBSS) and cells were incubated in RPMI, 10% FBS, 1% Pen-Strep, and 0.01% GM-CSF for 6 days. On day 6, the medium containing GM-CSF was removed, fresh RPMI was added, and cells were left for 24hrs before stimulation.

### 2.3 EV characterization

#### 2.3.1 EV isolation

Conditioned media from the basal chamber of PBEC cultures in ALI were filtered through 0.2 μM sterile filters (Sarstedt) and centrifuged at 10,000 g for 30 mins at 4°C to remove any larger particles and cell debris. The filtered conditioned media were then collected into 26.3 mL polycarbonate ultracentrifuge tubes (Beckman Coulter Life Sciences, Cat#355654) and centrifuged at 100,000 g for 3 hrs at 4°C. After ultracentrifugation, the EV-containing pellet was washed with 200 μL filter sterilized PBS (0.2 μm filters). The pellet was then resuspended in the appropriate medium for further analysis or experimental procedures. All EVs were analyzed per donor, technical replicates were averaged and shown as individual data points for each donor.

#### 2.3.2 Nanoparticle tracking analysis

The Malvern Panalytics NanoSight (model N300) was used throughout. Filter-sterilized PBS (500 μL) was always loaded and read first to ensure no contamination. EV isolates in PBS (500 μL total volume) were loaded into the chamber. The image focus and camera level were set to optimum conditions, and the detection threshold was set to contain 30–100 red crosses, deemed by the software as more valid readings, while maintaining 5 or less blue crosses per reading. Each sample was run for five 60s videos, and the mode of the particle size across the five videos recorded. Of note, PBS was recorded across three videos as the blank control before each set.

#### 2.3.3 Transmission electron microscopy

Formvar/carbon-coated copper grids (Agar Scientific) were incubated with 10 μL of EVs and stained with Uranyless (Electron Microscopy Sciences, Cat#22409). The grids with EVs were imaged using the TEM (TEM-JEM-1400Plus).

### 2.4 BCA

The total protein concentration of the samples was quantified using the Pierce BCA Protein Assay Kit (ThermoScientific) as per manufacturer’s instructions. The plate was read at 562 nm using the BioTek Synergy HTX Multimode Reader, and protein concentrations were calculated with reference to the standard curve.

### 2.5 EV-cell stimulation and inflammasome inhibition

Healthy and COPD EVs were diluted to 5 μg in 100 μL filter sterilized PBS (0.2 μm). Cells were washed with warmed PBS and the 100 μL EV-PBS solution was added. For cell-EV stimulation experiments, cells were topped up to 200 μL with the appropriate cell medium (supplemented with 1% FBS, 1% Pen-Strep). For inflammasome inhibition experiments, the NLRP3 inhibitor MCC950 and caspase inhibitor VX-765 were diluted to 2 μM and 40μ M respectively and the inhibitors were added to the cells at 100 μL inhibitor-RPMI into 100 μL EV-PBS solution, diluting MCC950 and VX-765 to the working concentrations of 1 μM and 20 μM respectively. Cells were then incubated at 37 °C, 5% CO_2_ for 48hrs. To note, any EV work was done in reduced serum media (1%) to limit significant FBS-EV contamination, while all untreated controls were also kept in reduced serum media on the same grounds (46).

### 2.6 Lactate dehydrogenase release

LDH release to determine cell viability was measured using the CyQUANT^TM^ LDH Cytotoxicity Assay Kit (Invitrogen) as per manufacturer’s instructions. The plate was read using the BioTek Synergy HTX Multimode Reader.

### 2.7 Enzyme-linked immunosorbent assay

Cytokine concentrations in cell media supernatants were analyzed using enzyme-linked immunosorbent assays (ELISAs) as per manufacturers’ instructions. The ELISA kits used are detailed in **Supplementary Table 3**. The plates were read using the BioTek Synergy HTX Multimode Reader.

### 2.8 Proteomic analysis

#### 2.8.1 Sample prep

Isolated EV pellets were resuspended in 8 M urea on ice. Samples were incubated with dithiothreitol (1 µl of 500 mM) and incubated at RT for 30 mins. Iodoacetamide (10 mM final concentration) was added to each sample and incubated at RT for 30 mins in the dark. Tris (pH 8.5, final concentration 2 M) and 1 µl of trypsin (0.5 µg/µL) was added to each sample. The tubes were placed at 37°C overnight in a shaking incubator. The digestion reaction was stopped after 18 hours by placing the tubes in -80°C.

#### 2.8.2 Mass spectrometry

All digested samples were analyzed in duplicate using the Bruker TimsTOF Pro mass spectrometer (MS) connected to an Evosep One chromatography system for detection. Tryptic peptides were resuspended in formic acid (0.01%) and loaded onto the Evosep One. The machine parameters remained constant throughout peptide separation – positive ion mode with capillary voltage of 1400 V, dry gas flow of 3 L/min, and a dry temperature of 180°C. The MS used Parallel Accumulation-Serial Fragmentation (PASEF) in trapped ion mobility spectrometer (TIMS) mode to select for ions by MS/MS. A scan range of 100–1700 m/z was performed at a rate of 10 PASEF MS/MS frames to 1MS scan with a cycle time of 1.8ws.

#### 2.8.3 MaxQuant protein identification and quantification

The raw MS data was then searched against the Homo sapiens subset of the Uniport Swissport database using the search engine MaxQuant (release 2.0.3.0) using specific parameters for trapped ion mobility spectra data-dependent acquisition (TIMS DDA). Only peptide scores that corresponded to a false discovery rate (FDR) of 0.01 were accepted from the MaxQuant database search. The normalized protein intensity of each identified protein was used for label free quantification (LFQ).

#### 2.8.4 Proteomic data analysis

Data were analyzed in R Studio (version 2023.06.0 + 421 “Mountain Hydrangea” Release) (47). All proteomic analysis was done using the DEP package (48). Firstly, any proteins with a unique peptide count of <1 were removed, and proteins with 2 or more missing values in each condition were also removed. Data was normalized and imputation was done to account for any other missing values (variance stabilization normalization and Quantile Regression Imputation of Left-Censored Data methods respectively). The Benjamini-Hochberg (BH) method was used to account for false discovery rate (FDR) (49). For differential analysis, p-values were set to 0.05, and log fold change at 1.5. Any proteins that fit both these parameters were run against GO Biological Processes 2023 to highlight potential functional roles using EnrichR software (50–52).

### 2.9 RNA sequencing

#### 2.9.1 RNA isolation and preparation

THP-1 cells were differentiated into macrophage-like cells using PMA and stimulated with COPD EVs +/- inflammasome inhibitors as described above. After 48 hrs, EV-stimulated THP-1 cells were detached with trypsin and pelleted (900 g for 5 mins at RT°). Cell pellets were sent to the Genomics Core Technology Unit at Queen’s University Belfast for RNA extraction and sequencing. The RNA extraction was done via the RNeasy® Plus using elution columns, as previously described (53).

#### 2.9.2 RNA sequencing data analysis

For RNA sequencing analysis, primary alignment was done through the Queen’s University Belfast Genomics Core Technology Unit and exported into Microsoft Excel. Differential gene expression were analyzed in R Studio (version 2023.06.0 + 421 “Mountain Hydrangea” Release) (47). All differential gene expression analysis was done using the DESeq2 package (54). Log fold change (LFC) shrinkage was done via the *apeglm* method (55). Data were organized into adjusted p-values and set to 0.05. Any genes that fit this parameter were deemed significantly differentially expressed.

### 2.10 Bacterial infection of macrophages

#### 2.10.1 *NTHi* strain and culture

*Non-Typeable Haemophilus influenzae* (*NTHi*) was used throughout infection experiments. The *NTHi* used was the genome sequenced clinical isolate NTHi375, kindly donated by Derek Hood from MRC, Harwell (Oxford, United Kingdom). *NTHi* was stored at -80 °C in cryovials in a 50% solution of glycerol. To recover *NTHi*, an inoculation loop was used to streak the semi-frozen stock onto chocolate agar. The plate was then incubated at 37°C overnight. After this incubation, a single colony was taken from the agar and suspended in BHI supplemented with heparin and NADH at 37°C overnight. The following morning, 500µL of the bacteria-containing broth was diluted in 20mL fresh BHI and incubated again at 37°C for 3.5hrs to ensure bacteria were in the exponential growth phase when cells were infected.

#### 2.10.2 Macrophage stimulation with *NTHi*

PMA-differentiated THP-1 macrophages were stimulated with COPD or healthy EVs (5 µg/well) 30 minutes prior to infection. Cells were incubated with *NTHi* (MOI 10) at 37°C, 5% CO_2_ for 2 hrs, after which cells were lysed using 0.1% Triton X. Cell lysates were plated onto chocolate agar and incubated overnight at 37°C. After 24 hrs, surviving colonies were counted and CFUs calculated. For ELISA analysis, the supernatants of the remaining cells that had not been lysed were removed and replaced with 100 mg/mL RPMI supplemented with gentamycin for 1 hr. After 1 hr, the media was replaced with RPMI supplemented with 25 mg/mL gentamycin and left for 22 hrs. After this time, plates were centrifuged at 500 g for 5 mins at RT°, supernatants were collected and used for cytokine analysis.

### 2.11 Statistical analysis

Data was primarily handled within Microsoft Excel. All analyses of data were done in GraphPad Prism Version 10.4.1 (GraphPad Software Inc, San Diego, CA) and are reported as mean ± SEM. Data were tested for normality by the Shapiro-Wilk test to determine whether parametric or non-parametric tests were to be used for statistical analysis. Data were considered significant if *p* < 0.05. Statistical significance was indicated as follows: * *p* < 0.05, ** *p* < 0.01, *** *p* < 0.001, and **** *p* < 0.0001. Any data that did not reach statistical significance was labelled ns (not significant).

## 3 Results

### 3.1 Characterization of healthy and COPD epithelial EVs

**Figure 1:**
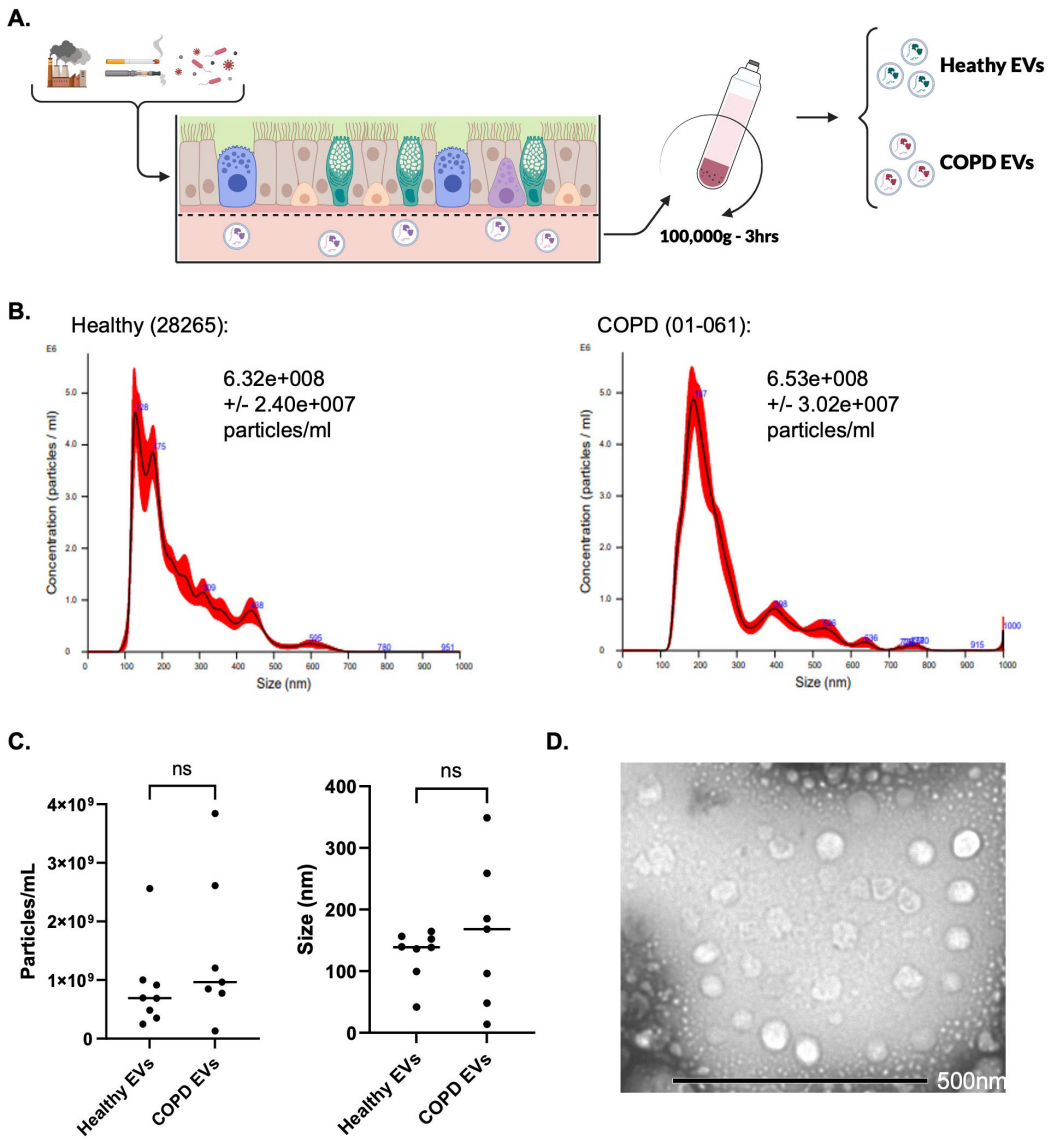
Isolation and characterization of epithelial extracellular vesicles. **(A)** Schematic of ALI cell culture and subsequent isolation of basal chamber EVs by ultracentrifugation. **(B)** Examples of the size and concentration distribution of epithelial cell-derived EVs isolated from the basal chamber of healthy and COPD donor cells in ALI. Histograms were generated from five 60s videos/sample. **(C)** Mode size (nm) of particles in isolation for each donor, and average concentration in particles/mL in isolation for each donor (n=8 healthy donors, n=7 COPD donors). Data were analyzed by Mann-Whitney test. Statistical significance was indicated as follows: P values > 0.05 were indicated using ns (not significant). **(D)** Representative TEM images to show spherical morphology of epithelial cell-derived EVs. Images are representative of four independent donors at a magnification of x25k. Scale bar – 500nm.

Healthy epithelial EVs (HepEVs) and COPD epithelial EVs (CepEVs) were isolated from the basal aspect of PBEC ALI cultures (**Figure 1A**) and analyzed by NTA (**Figure 1B**). HepEVs and CepEVs did not significantly differ in either size or concentration (**Figure 1C**). Since NTA provides information on size and concentration of particles in suspension, but cannot conclusively confirm these particles are indeed EVs, we further validated the presence of EVs by TEM. The darker outer membrane and ‘cup-shaped’ morphology of EVs was evident by TEM (**Figure 1D**).

### 3.2 CepEV-stimulation promotes inflammasome gene expression in macrophages

**Figure 2:**
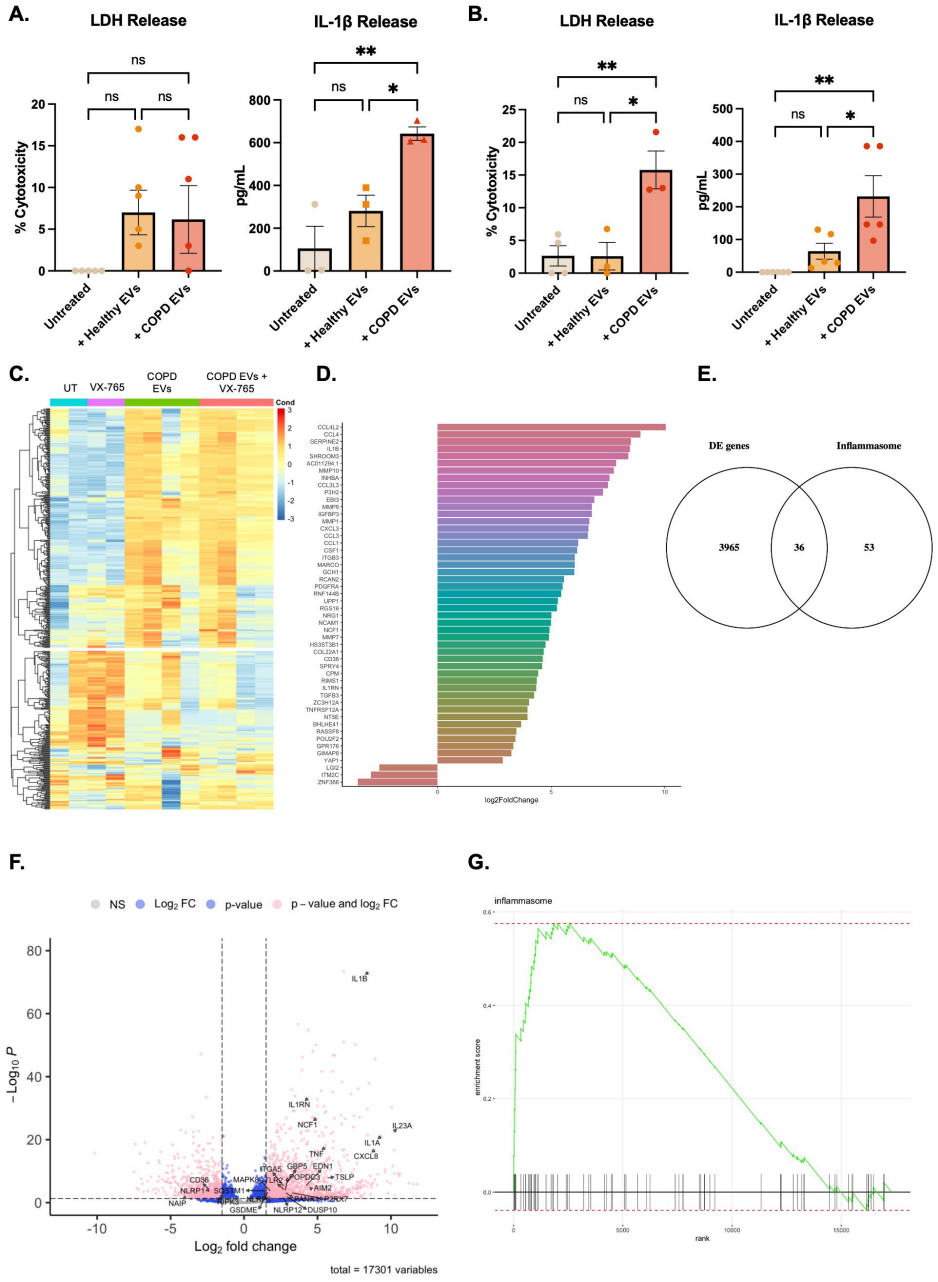
RNA sequencing of CepEV-stimulated THP-1 cells. **(A)** Cell viability in THP-1 cells stimulated with 5µg EVs was determined via LDH release. All LDH release was compared to a 100% lysed control and expressed as percentage cytotoxicity (n=5 technical replicates of five donors). Data were analyzed by Kruskal-Wallis test with Dunn’s multiple comparisons test. The level of IL-1β release in THP-1 cells stimulated with 5µg EVs were quantified by ELISA (n=3 technical replicates of five donors). Data were analyzed by one-way ANOVA with Dunnett’s multiple comparisons test. **(B)** Cell viability in MDMs stimulated with 5µg EVs was determined via LDH release. All LDH release was compared to a 100% lysed control and expressed as percentage cytotoxicity (n=3 technical replicates of three donors). The level of IL-1β release in MDMs stimulated with 5µg EVs were quantified by ELISA (n=3 technical replicates of five donors). Both were analyzed by Kruskal-Wallis test with Dunn’s multiple comparisons test. **(C)** A heatmap showing the top 500 differentially expressed genes with a significant p-value (<0.05) in THP-1 cells under various conditions. Hierarchical clustering was done on rows (proteins), but not in columns (conditions). Color key for conditions are as follows: Blue – Untreated, purple – VX-765, green – COPD EV stimulated, and red – COPD EV stimulated + VX-765. **(D)** A bar graph of the top 50 significantly (<0.05 p value) differentially expressed genes, ordered by log foldchange. Package used to construct the bar graph was ggplot2. **(E)** Venn diagram of the 4001 differentially expressed genes, labelled DE genes, against the Harmonizome 3.0 list of inflammasome associated genes, labelled inflammasome. Venn diagram was constructed using Venny (56). **(F)** Volcano plot of the 17,301 genes identified, 4001 of which were significantly differentially expressed in COPD EV stimulated cells. Dashed lines indicate cutoff values set, 1.3 on the y-axis (corresponding to a p-value of <0.05), and 1 on the x-axis (corresponding to a fold change of >2). Package used to construct the volcano plot was Enhancedvolcano version 1.14.0 (57). **(G)** GSEA enrichment plot for inflammasome signature, comparing untreated THP-1 cells to COPD epithelial cell-derived EV stimulated THP-1 cells. The Harmonizome 3.0 inflammasome gene list was used to conduct GSEA. Statistical significance was indicated as follows: *P < 0.05. Any P values > 0.05 were indicated using ns (not significant).

To assess the functional impact of epEV exposure in macrophages, THP-1 cells (**Figure 2A**) and primary monocyte-derived macrophages (MDMs) (**Figure 2B**) were stimulated with healthy and COPD epEVs and assessed for LDH release as a measure of cytotoxicity and IL-1β release as a measure of inflammasome activation. Compared to untreated and HepEV exposure conditions, CepEV exposure increased IL-1β release in both THP-1 and MDMs. This effect was associated with increased LDH release in MDMs, but not THP-1 cells. By contrast, HepEV exposure compared to untreated conditions did not increase IL-1β release, suggesting a CepEV-specific response.

To delve further into the mechanism behind the pro-inflammatory cytokine release and cell death seen with CepEV stimulation, RNA sequencing of PMA-differentiated THP-1 cells stimulated with CepEVs from various donors was performed. As we expected inflammasome activation to play a role due to increased IL-1β release, we included VX-765, a potent caspase inhibitor, as a control. Analysis of RNA sequencing data uncovered 4,001 differentially expressed genes (DEGs) in THP-1 cells stimulated with CepEVs when compared to HepEVs. A heatmap was first constructed of the top 500 differentially expressed genes in this data set (**Figure 2C**). Hierarchical clustering was performed on the genes which highlights up- and down-regulated transcripts in CepEV-stimulated cells, after which the list of DEGs was narrowed down to the top 50, and ordered by log fold-change (**Figure 2D**). The majority (47) of the top 50 DEGs was upregulated by CepEV stimulation, with only 3 being downregulated. When compared against the Harmonizome 3.0 list of inflammasome-associated genes (58, 59), we found there was overlap with 36 genes in our list of DEGs (**Figure 2E**). Next, a volcano plot was constructed (**Figure 2F**) for all 17,301 genes detected in our analysis. Data points in blue cross one threshold (p value or log fold-change), while those in pink are genes that cross both thresholds and therefore are significantly differentially expressed. Any inflammasome genes from the Harmonizome that met both parameters are labelled. After highlighting that various inflammasome-associated genes were differentially expressed after CepEV stimulation, a specific gene set enrichment analysis (GSEA) was performed to assess the association with the inflammasome (**Figure 2G**). The list of inflammasome-associated genes was taken from the Harmonizome 3.0 list used for the previous results.

### 3.3 Inflammasome inhibition reduces IL-1β release by CepEV-stimulated macrophages

**Figure 3:**
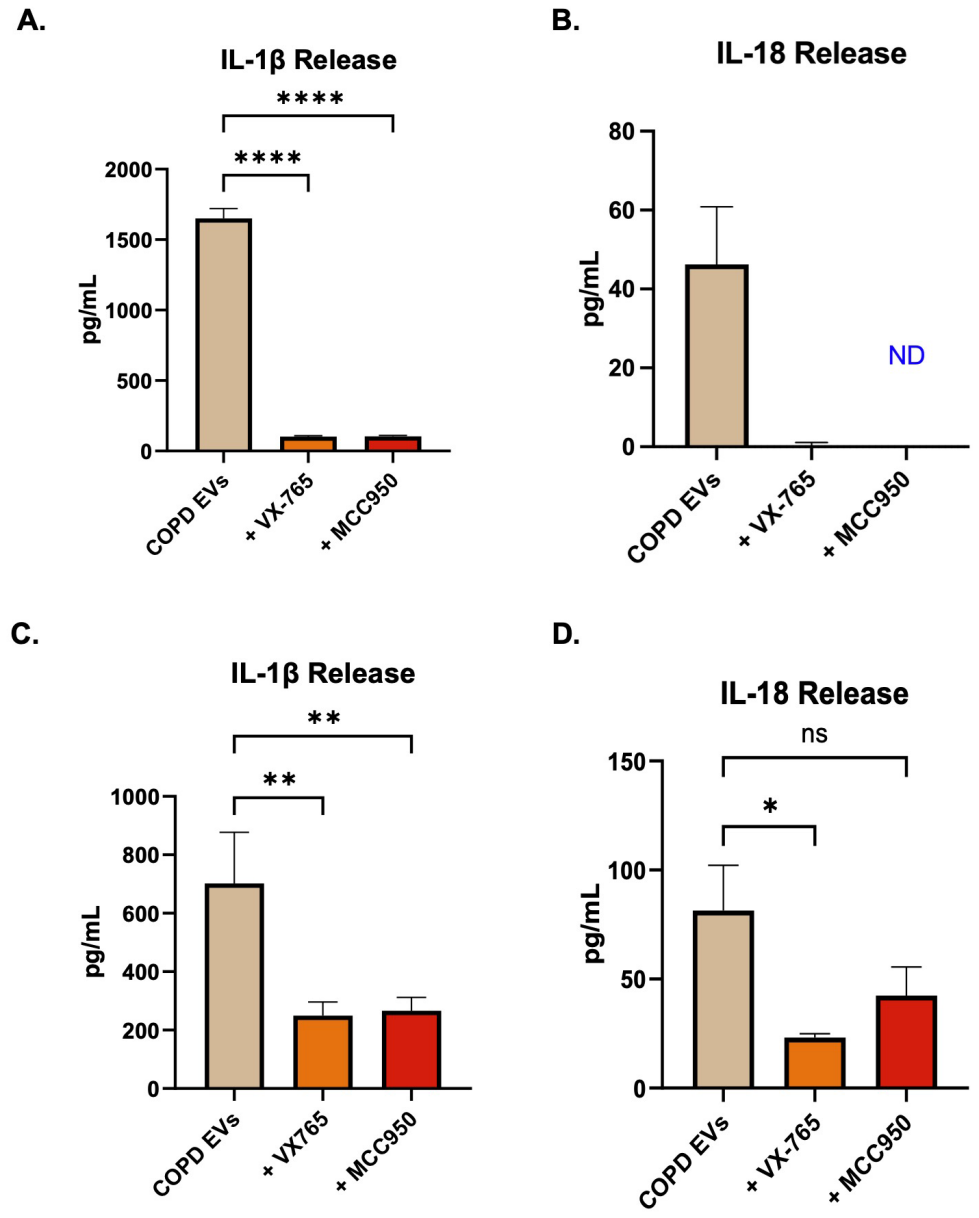
Inflammasome inhibition in CepEV-stimulated macrophages. **(A, B)** The levels of IL-1β and IL-18 release in THP-1 cells stimulated with 5µg EVs +/- inflammasome inhibitors were quantified by ELISA (n=3 technical replicates of three donors for each cytokine). Cells stimulated with EVs and treated with MCC950 were labelled ND for ‘not detectable’ for IL-18 as the values were below the ELISA kit minimum detection threshold. **(C, D)** The level of IL-1β and IL-18 release in MDMs stimulated with 5µg EVs +/- inflammasome inhibitors were quantified by ELISA (n=6). THP-1 data and MDM IL-1β were analyzed by one-way ANOVA with Dunnett’s multiple comparisons test, MDM IL-18 by Kruskal-Wallis test with Dunn’s multiple comparisons test. Statistical significance was indicated as follows: *P < 0.05, **P < 0.01, ****P < 0.0001.

After showing that there was significant IL-1β release both in THP-1 cells and MDMs after CepEV stimulation, and confirming the up-regulation of various inflammasome genes in THP-1 cells, we sought to confirm inflammasome activation. First, we set out to test the effects of the potent NLRP3 inflammasome inhibitor MCC950, and caspase inhibitor VX-765, on THP-1 cells stimulated with CepEVs. The release of IL-1β in THP-1 cells stimulated with CepEVs was reduced significantly by the addition of either VX-765 or MCC950 (**Figure 3A**). As an independent outcome, another inflammasome-associated cytokine, IL-18, was assessed. Again, VX-765 and MCC950 reduced the levels of IL-18 induced after CepEV stimulation (**Figure 3B**). These findings were confirmed in primary MDMs, in which the release of both IL-1β (**Figure 3C**) and IL-18 (**Figure 3D**) release were decreased by VX-765. However, while NLRP3 inflammasome inhibition using MCC950 significantly reduced IL-1β release, IL-18 release although strongly reduced was not statistically significant.

### 3.4 Proteomic analysis of CepEVs highlights absence of antibacterial proteins

EVs have a complex role in infections. It has previously been shown in multiple studies with various bacteria that infection can influence the content of EVs released from infected host cells. These EVs can result in either cell survival or cell death, inflammation and an immune response, or allow the pathogen to survive within the host. Research into the role of EVs and infection has focused on the release of EVs from infected cells, their altered content, and the effect this has on surrounding cells. To date, the potential for EVs derived from uninfected diseased cells to elicit and alter responses to infection has not been determined. Initially, we set out to explore the protein content of CepEVs from COPD donors and compare it to that of HepEVs from age-matched controls to explore mechanisms of disease, including a potential role in enabling bacterial infection. To this end, CepEVs and HepEVs were isolated from epithelial ALI cultures and untargeted label-free quantification was carried out to obtain the relative abundance of proteins in each sample.

**Figure 4:**
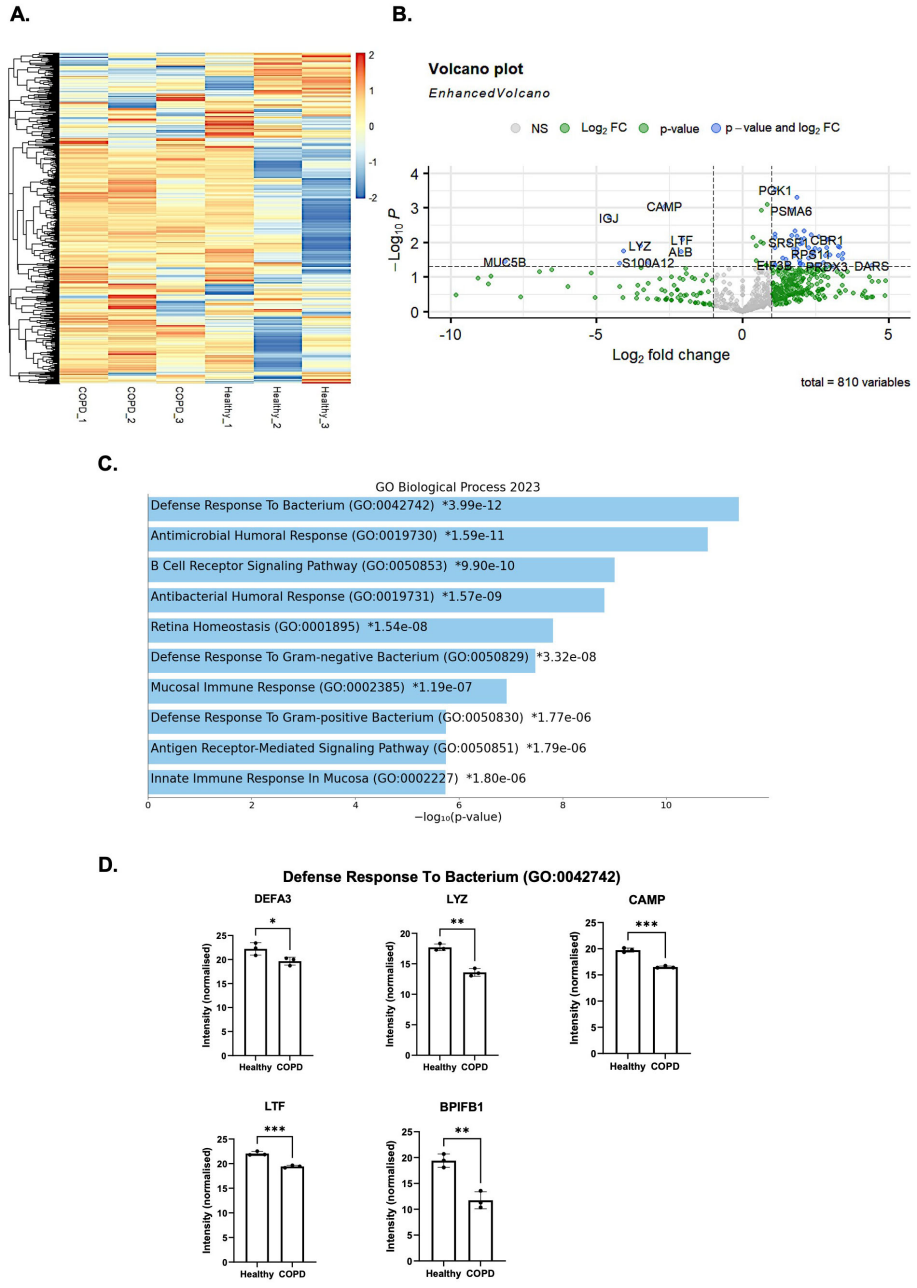
Proteomic analysis of HepEVs and CepEVs. **(A)** A heatmap of proteins differentially expressed with a significant p-value (0.05). Hierarchical clustering was done on rows (proteins), but not in columns (conditions). **(B)** Volcano plot of the proteome of healthy and COPD EVs identified 810 proteins in total. Dashed lines indicate cutoff values set, 1.3 on the y-axis (corresponding to a p-value of <0.05), and 1 on the x-axis (corresponding to a fold change of >2). **(C)** Proteins were compared against the 2023 database of GO Biological Process terms. The significance of these pathways are highlighted by an * **(D)** The proteins plotted are: defensin alpha 3 (DEFA3), lysozyme (LYZ), cathelicidin antimicrobial peptide (CAMP), lactotransferrin (LTF), and BPI fold containing family B member 1 (BPIFB1). Data were analyzed by paired t-test. Statistical significance was indicated as follows: *P < 0.05, **P < 0.01, ***P < 0.001. Any P values > 0.05 were indicated using ns (not significant).

After initial quality controls and normalization procedures, 810 proteins were identified in the EV samples. To explore differences between HepEVs and CepEVs, a heatmap was constructed of proteins that were differentially expressed between the two (**Figure 4A**). Although these proteins had been selected as being significantly expressed between conditions, the heatmap did not easily display these changes. We next generated a volcano plot, which allowed us to more easily visualize differentially enriched proteins between the two groups (**Figure 4B**). We split the differentially enriched proteins into two sets, respectively showing proteins identified at significantly higher or lower abundance in CepEVs vs. HepEVs. We performed pathway analysis and plotted the GO terms most associated with each set and observed that the top 10 GO Biological Processes associated with the proteins present at a lower abundance in CepEVs were almost all related to antibacterial mechanisms (**Figure 4C**). The top GO term was ‘Defense Response to Bacterium’; **Figure 4D** shows the differentially enriched proteins associated with this term. The lower abundance proteins identified through this analysis, alongside the corresponding fold changes (FC) are outlined below: Defensin alpha 3 (DEFA3) [-2.028] – a protein identified in neutrophil granules, lysozyme (LYZ) [-3.503] – protein with bacteriolytic function, cathelicidin antimicrobial peptide (CAMP) [-2.659] – an antimicrobial protein involved in immune modulation, Lactotransferrin (LTF) [-2.066] – a multifunctional protein able to sequester iron from bacteria to inhibit growth, and BPI fold containing family B member 1 (BPIFB1) [-6.957] – a protein with the ability to bind to and neutralize LPS.

### 3.5 CepEV exposure reduces the ability of macrophages to clear *NTHi* infection

**Figure 5:**
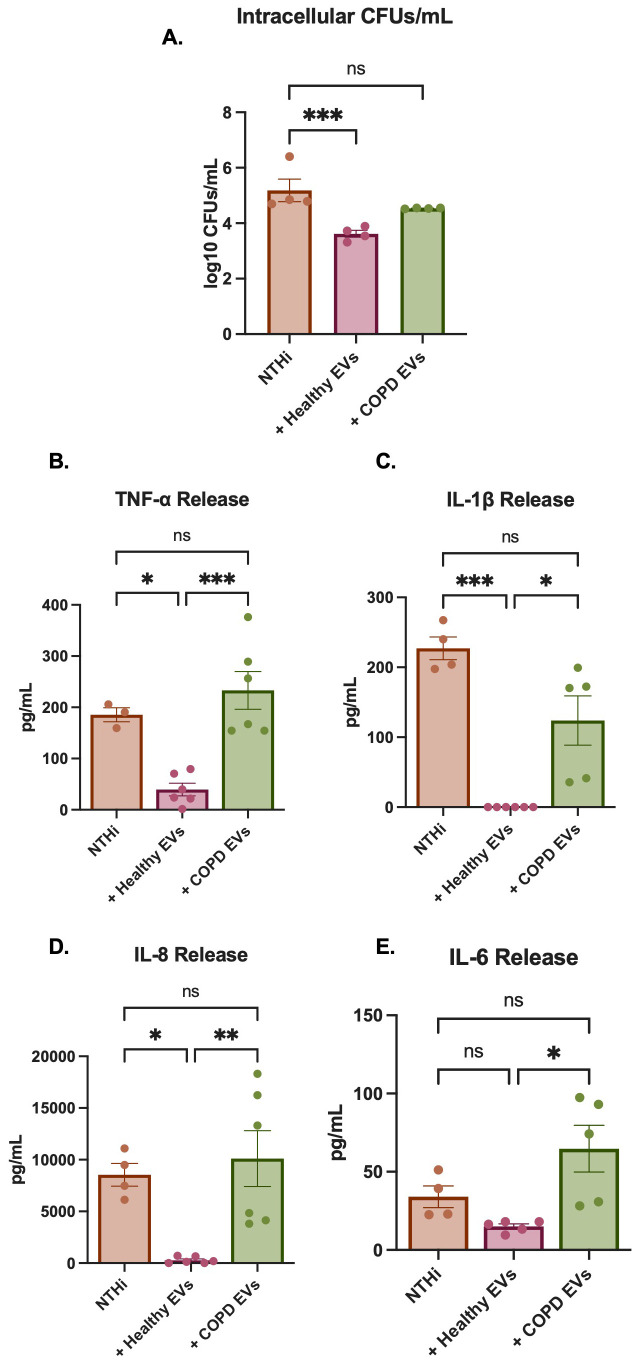
NTHi infection in CepEV-stimulated THP-1 cells. **(A)** THP-1 cells were pre-incubated with 5μg EVs (n=5 for COPD donors, n=4 for healthy donors), then infected with NTHi at MOI10. After 2hrs cells were lysed and plated on chocolate agar overnight at 37°C. The number of colony-forming units (CFUs) were calculated for each plate and expressed as the logarithm of the number of CFUs/mL. Data shown is the result of 3 technical replicates for four EV donors. Data were analyzed by one-way ANOVA with Dunnett’s multiple comparisons test. **(B–E)** The release of TNF-α (n=6 donors), IL-1β (n=5 donors), IL-8 (n=6 donors) and IL-6 (n=5 donors) were quantified by ELISA. Data were analyzed by one-way ANOVA with Dunnett’s multiple comparisons test. Statistical significance was indicated as follows: *P < 0.05, **P < 0.01, ***P < 0.001. Any P values > 0.05 were indicated using ns (not significant).

After proteomic analysis of CepEVs showed significantly reduced levels of various proteins involved in antibacterial responses, we moved to assess the functional impact of these EVs during an active bacterial infection. As *NTHi* is the most common cause of infection in COPD patients, we used this bacterium to assess the function of EVs. To assess THP-1 cell susceptibility to infection after pre-incubation with EVs, cells were infected for 2 h, then lysed and intracellular colony-forming units (CFUs) calculated (**Figure 5A**). As shown, pre-incubating THP-1 cells with HepEVs reduced intracellular CFUs significantly, suggesting HepEVs promoted bacterial clearance, while this mechanism was lost when THP-1 cells were incubated with CepEVs. Of note, there was no change in extracellular CFUs in infected cells pre-incubated with HepEVs or CepEVs as compared to infected cells alone. This suggests that HepEVs promote the intracellular killing, rather than internalization, of *NTHi* in THP-1 cells.

We next measured the release of inflammatory cytokines in *NTHi*-infected THP-1 cells under HepEV and CepEV exposures. Supernatants were collected 24 hrs after infection and levels of four pro-inflammatory cytokines (TNF-α, IL-1β, IL-8, and IL-6) associated with infection were assessed. As a control, we ensured that the levels of all pro-inflammatory cytokines assessed were significantly increased in *NTHi* infected THP-1 cells compared to uninfected cells (**Supplementary Table 4**). As shown in **Figure 5B–E**, pre-incubation of THP-1 cells with HepEVs significantly reduced TNF-α, IL-1β, and IL-8 release post-*NTHi* infection. Pre-incubation with CepEVs had no significant effect on pro-inflammatory cytokine release compared to *NTHi* infection alone, again suggesting that a key mechanism of host antibacterial defense is defective in macrophages exposed to EVs from COPD epithelium.

## 4 Discussion

### 4.1 Inflammasome priming and activation

RNA sequencing was conducted in this study to uncover the effect that epithelial cell-derived EVs have on macrophages at the transcriptional level. An interesting finding in this study was that CepEV stimulation promoted upregulation of various inflammasome-associated genes, and induced IL-1β release, seemingly both priming and activating the inflammasome without other external stimuli. It is important to note that a significant portion of COPD, and COPD-derived EV studies have focused on the role of miRNAs, which requires a specialized type of sequencing known as small RNA sequencing (60–64). While this is essential for exploring the content of EVs, our goal here was to investigate the effect of the EVs on the cells, so bulk RNA sequencing was used. In conjunction with this, known inflammasome inhibitors were able to resolve the release of IL-1β from CepEV-stimulated macrophages, highlighting the inflammasome as a key feature of the epithelial-macrophage inflammatory cross-talk in COPD.

The coordination of tissue activity in a complex organ like the lung is contingent on cell-to-cell communication between neighboring cells of the same type, and across cell types. While bronchial epithelial cells are a vital first line of defense against inhaled irritants that can initiate COPD pathogenesis, macrophages are also known to play a key role in this process, notably in relation to pro-inflammatory signaling and epithelial remodeling. EVs are key mediators in the crosstalk between cell types (65). Specifically, EVs released from the respiratory epithelium have previously been shown to alter macrophage inflammatory signaling (43). EVs from cigarette smoke extract-treated epithelial cells have been shown to promote macrophage polarization and pyroptosis in a COPD mouse model, both of which are key players in COPD progression (66).

An interesting feature of this study is that the CepEVs were able to seemingly prime and activate the inflammasome without other external stimuli, despite this commonly being a two-step process (67). The ability of EVs to act as priming and activating stimuli has been shown previously, however not in the context of epithelial cells in COPD. Both priming and activation of the inflammasome can occur in response to EVs generated from a methicillin-resistant strain of *Staphylococcus aureus* bacterium (MRSA) (68). In that study, it was shown that lipoproteins on the surface of the bacterial EVs, or outer-membrane vesicles (OMVs) as they are referred to in the context of bacteria, were able to prime the inflammasome through TLR2 signaling. In addition, pore-forming toxins within OMVs were able to induce inflammasome activation through K+ efflux. Another study detected this effect in the CF lung. CF neutrophil-derived EVs have been shown to induced upregulation of caspase-1, alongside IL-1β and IL-18 extracellular release in epithelial cells (69). Interestingly, it has also been shown that epithelial cells in the CF lung can activate neutrophils and regulate neutrophil chemotaxis (70). While the latter of these studies was not inflammasome activation-specific, it is important to note for our study that signaling between cells can be multidirectional, and we were able to show that epithelial cell-derived EVs were able to induce inflammasome activation in macrophages. It would be important to further confirm these findings in primary MDMs.

VX-765 is a small molecule inhibitor that works to competitively inhibit caspase-1 (71, 72). MCC950 is regarded as the most specific NLRP3 inflammasome inhibitor to date, inhibiting NLRP3-induced ASC oligomerization and subsequent caspase-1 cleavage (73, 74). Both of these well-established inflammasome inhibitors reduced CepEV-induced IL-1β release in THP-1 cells and primary MDMs, confirming that NLRP3 inflammasome was the driving force in the observed IL-1β release. This is a key finding of our study, as to date it has not previously been shown that epithelial EVs from COPD patients drive inflammasome activation in macrophages. It should also be noted that, despite a significant reduction in both IL-1β and IL-18 release with VX-765 and MCC950 treatment, these inhibitors were not able to fully block cytokine release in THP-1 cells or primary MDMs. As our experimental design included stimulation of macrophages for 48 hrs, it is possible that the remaining IL-1β and IL-18 signal detected after inhibition could be the result of the inhibitors being fully bound or consumed. An additional dose of each inhibitor at the experiment midpoint (24 hrs post-EV stimulation) may be tested in future studies. Increasing the concentration of inhibitor used may also reduce IL-1β and IL-18 levels further, however the concentrations chosen had been previously tested in our lab on the cell types studied, and an increase in concentration could lead to reduced cell viability. We note that it not unusual for studies to report a decrease in inflammasome-associated cytokines, rather than complete inhibition (75, 76).

### 4.2 Macrophage *NTHi* infection

Uncovering the proteome of CepEVs could generate significant insight into COPD pathogenesis. Indeed, findings of this study highlighted potential roles of CepEVs in facilitating bacterial infections. CepEVs were found to have a significantly lower abundance of a variety of proteins with known antibacterial roles. After functionally assessing the effect of CepEVs in the context of macrophage *NTHi* infection, we were able to show that macrophages stimulated with CepEVs, as oppose to HepEVs, were unable to clear *NTHi* or mediate the inflammatory burden of bacterial infection.

Proteins are increasingly recognized as critical players in EV-mediated intracellular communication, as revealed by proteomic analysis of EVs in the context of cardiovascular disease, Parkinson’s, and rheumatoid arthritis (77–79). Recurrent bacterial and viral infections are a key feature of COPD, and are now clearly linked with the occurrence of acute exacerbations, increased airway inflammation and faster disease progression in patients (80). The most common bacterial infection observed in the lungs of patients with COPD is *NTHi* (81). To date, the role of COPD epithelial EVs and age-matched controls had not been studied in the context of *NTHi* infection of macrophages. Amongst the proteins identified during proteomic analysis that were decreased in CepEVs were DEFA3, LYZ, CAMP, LTF, and BPIFB1, all of which had strong associations with defense response to bacterium (GO: 0042742), as well as the immune response to bacterial infection (82, 83). Interestingly, of these five proteins identified for their antibacterial roles, two are members of the main classes of mammalian antimicrobial peptides (AMPs), namely defensins and cathelicidins (84). AMPs provide a non-specific, rapid response to bacteria, and have been shown to have a potent bactericidal effect on both Gram-positive and negative bacteria (85). DEFA3 is an α-defensin generally involved in host defense (86). Interestingly, it has been shown that α-defensins can specifically enhance the adherence of *NTHi* to MDMs, and that this increased adherence promotes TNF-α expression (87). As we observed a decrease in TNF-α release, alongside no change in extracellular CFUs, our data suggests that DEFA3 may not be involved in the mechanism(s) of bacterial clearance at play in our study. CAMP is a member of the cathelicidin AMP subgroup. To date, there has only been a single identified cathelicidin in humans, LL-37 (88). LL-37 can directly kill bacteria through membrane disruption by binding to negatively charges on their membrane, leading to the generation of pores that cause a loss of cell integrity, lysis and death (89). It has also previously been shown that LL-37 can modulate gene expression in macrophages, thus influencing cell functions including chemokine and chemokine signaling (90, 91). This is consistent with our cytokine data, suggesting that LL-37 may work to promote bacterial clearance while minimizing the inflammatory effects, the effects of which were missing in the context of CepEV exposure. LTF (lactotransferrin) is a multifunctional iron-binding protein abundant in airway secretions, and acts as a first line of defense protein against inhaled pathogens (92–95). This protein is hugely significant in various respiratory infections, and interestingly, its effects can be modulated by cigarette smoke (96). Currently, there is little data about the role of LTF in *NTHi* respiratory infection, and our findings warrant further study to elucidate if LTF is indeed significantly involved in the clearance of *NTHi*. Finally, BPIFB 1 (bacteria permeability increasing protein fold containing family B member 1) is a secretory protein produced in the respiratory tract, and while its function is not fully understood, it is assumed that it plays a role in host defense (97). It has also been shown that COPD airways have elevated levels of BPIFB1 when compared to asymptomatic controls, and overexpression of BPIFB1 in macrophages is thought to promote pulmonary fibrosis and impair lung function (98–100). It is unclear that BPIFB1 is related to the loss of bacterial clearance in CepEV stimulated macrophages as there is no current evidence to suggest it is directly bactericidal. While out of the scope for this study, it would be important to study this protein further in the context of COPD as it may play a complex and as yet undefined role in pathogenesis.

Like most bacterial infections, *NTHi* infection induces the release of a variety of cytokines and chemokines, the dysregulation of which leads to airway fibrosis and immune cell influx, further propagating inflammation and tissue damage (101) An interesting result of this study was that incubation with healthy EVs significantly reduced the release of various inflammatory cytokines in response to *NTHi* infection in THP-1 cells (**Figure 5B**). In parallel with this reduction from healthy EVs, incubating THP-1 cells with COPD EVs had no significant effect on inflammatory cytokine resolution. IL-8 and IL-1β are strong neutrophil chemoattractants. Neutrophilic inflammation in people with COPD has shown increased risk of future exacerbations (102). However, while neutrophil influx has been linked to worse disease prognosis, neutrophils are significant contributors in the clearance of bacterial infections such as *NTH*i (103). This double-edged sword of neutrophil inflammation highlights how important the efficient and tight regulation of chemoattractants such as IL-8 and IL-1β are in response to bacterial infections, a regulatory mechanism that is lost in the presence of CepEVs. IL-6 is strongly linked with disease severity, exacerbation rates, and reduced lung function in people with COPD (104, 105). Although HepEVs did not significantly reduce the release of IL-6 compared to *NTHi* infected controls, CepEVs did significantly increase this release compared to HepEVs, suggesting CepEVs further exacerbate the release of IL-6 in response to *NTHi* infection.

In future studies, we will attempt to identify which of these key proteins is the most important for supporting the antibacterial activity against *NTHi* observed in macrophages treated with HepEVs. In addition to identifying specific protein targets, it will be interesting to assess if the same results hold with Gram-positive bacteria, or with other Gram-negative bacteria. While *NTHi* was chosen for this work due to its prevalence in COPD, *M. catarrhalis* and *S. pneumoniae* are also common bacterial pathogens in COPD, and in more advanced disease *P. aeruginosa* becomes more prevalent (106). As discussed above, LYZ (lysozyme) was a protein identified in the proteomic analysis of epithelial EVs that works predominantly to neutralize Gram-positive bacteria. Assessing the effect of these EVs in Gram-positive bacteria could further define the potential role of LYZ present in epithelial EVs. Moving forward, the ability of HepEVs to modulate THP-1 cell function and promote bacterial clearance will also need to be validated in primary macrophages. Also, a minimum inhibitory concentration assay (MIC) in the context EV co-incubation with *NTHi* will be necessary to assess *NTHi* growth, and whether the EVs have intrinsic antibacterial properties. We note however that we evaluated extracellular CFUs and observed no change between groups, regardless of the absence or presence of EVs (from COPD or healthy epithelia). This suggests that, at least in these experimental conditions, EVs were not significantly interacting with the bacteria outside of the cell or else extracellular CFUs would be reduced in EV-stimulated conditions, independent of cellular uptake.

The process of aging leads to a progressive decline in immune system function, even in healthy individuals (107). Interestingly, the higher average age of our healthy controls compared to our COPD cohort did not seem to have any effect on the antibacterial abilities of the healthy EVs, as pre-incubation with each of our HepEV donors resulted in nearly a complete elimination of IL-1β and IL-8 signal (**Figure 5B**). Our results were consistent amongst age groups tested, providing evidence to suggest that this mechanism of bacterial clearance is conserved and not lost with age. We also only had one female donor in our healthy cohort, and although we saw no differences in the response in this donor, we would be unable to conclude that there is no sex difference in this mechanism without assessing more female donors recruited to the study.

It has not previously been shown that CepEVs drive inflammasome activation in healthy macrophages, and prompts the question of inflammasome inhibition as an avenue for COPD therapeutics. While VX-765 has been widely used in *in vitro* and in animal model studies in a variety of diseases (108–111), phase II clinical trials into the treatment of inflammatory conditions have so far been unsuccessful due to liver toxicity (112). MCC950 is also in phase II clinical trials for rheumatoid arthritis (RA), however increased serum liver enzyme levels were detected, again suggesting liver toxicity, halting the trial (113). While the use of both VX-765 and MCC950 have not yet progressed beyond phase II clinical trials, the effectiveness of their use in this study cannot be overlooked. The use of EVs themselves also offers the potential for various therapeutic options. EVs, being naturally occurring carriers of molecular content, are less likely to induce a detrimental immune response compared to an artificial delivery system (7). This work highlights the significant reduction in antibacterial proteins needed to aid macrophage clearance of *NTHi*. By the addition of missing antibacterial proteins, either through the administration of healthy, antibacterial protein-rich EVs, or through the modification and supplementation of these missing proteins (described in detail elsewhere (114–116)) patients with COPD could see a significant reduction in bacterial burden of disease-progressing bacteria such as *NTH*i.

Taken together, this study establishes a significant effect of COPD epithelium-derived EVs on inflammasome signaling by macrophages and on their antibacterial response to *NTHi*, which may influence the chronic and acute course of inflammation and infection in patients and open potential avenues for therapeutic intervention.

## Data Availability

The data that supports the findings of this study have been deposited in NCBI’s Gene Expression Omnibus (GEO) repository, and are accessible through GEO Series accession number GSE313809 (https://www.ncbi.nlm.nih.gov/geo/query/acc.cgi?acc=GSE313809).

## References

[1] Robinson DG Ding Y Jiang L . Unconventional protein secretion in plants: a critical assessment. Protoplasma. (2016) 253:31–43. doi: 10.1007/s00709-015-0887-1, PMID: 26410830

[2] Liu C Yazdani N Moran CS Salomon C Seneviratne CJ Ivanovski S . Unveiling clinical applications of bacterial extracellular vesicles as natural nanomaterials in disease diagnosis and therapeutics. Acta Biomater. (2024) 180:18–45. doi: 10.1016/j.actbio.2024.04.022, PMID: 38641182

[3] Burgelman M Vandendriessche C Vandenbroucke RE . Extracellular vesicles: A double-edged sword in sepsis. Pharmaceuticals. (2021) 14:829. doi: 10.3390/ph14080829, PMID: 34451925 PMC8399948

[4] Mazzeo C Cañas JA Zafra MP Rojas Marco A Fernández-Nieto M Sanz V . Exosome secretion by eosinophils: A possible role in asthma pathogenesis. J Allergy Clin Immunol. (2015) 135:1603–13. doi: 10.1016/j.jaci.2014.11.026, PMID: 25617225

[5] Cañas JA Sastre B Mazzeo C Fernández-Nieto M Rodrigo-Muñoz JM González-Guerra A . Exosomes from eosinophils autoregulate and promote eosinophil functions. J Leukoc Biol. (2017) 101:1191–9. doi: 10.1189/jlb.3AB0516-233RR, PMID: 28096299

[6] Holtzman J Lee H . Emerging role of extracellular vesicles in the respiratory system. Exp Mol Med. (2020) 52:887–95. doi: 10.1038/s12276-020-0450-9, PMID: 32541816 PMC7338515

[7] Trappe A Donnelly SC McNally P Coppinger JA . Role of extracellular vesicles in chronic lung disease. Thorax. (2021) 76:1047–56. doi: 10.1136/thoraxjnl-2020-216370, PMID: 33712504 PMC8461402

[8] Hough KP Wilson LS Trevor JL Strenkowski JG Maina N Kim YI . Unique lipid signatures of extracellular vesicles from the airways of asthmatics. Sci Rep. (2018) 8:10340. doi: 10.1038/s41598-018-28655-9, PMID: 29985427 PMC6037776

[9] Adeloye D Song P Zhu Y Campbell H Sheikh A Rudan I . Global, regional, and national prevalence of, and risk factors for, chronic obstructive pulmonary disease (COPD) in 2019: a systematic review and modelling analysis. Lancet Respir Med. (2022) 10:447–58. doi: 10.1016/S2213-2600(21)00511-7, PMID: 35279265 PMC9050565

[10] Bhatta DN Glantz SA . Association of E-cigarette use with respiratory disease among adults: A longitudinal analysis. Am J Prev Med. (2020) 58:182–90. doi: 10.1016/j.amepre.2019.07.028, PMID: 31859175 PMC6981012

[11] Al Wachami N Guennouni M Iderdar Y Boumendil K Arraji M Mourajid Y . Estimating the global prevalence of chronic obstructive pulmonary disease (COPD): a systematic review and meta-analysis. BMC Public Health. (2024) 24:297. doi: 10.1186/s12889-024-17686-9, PMID: 38273271 PMC10811845

[12] Boers E Barrett M Su JG Benjafield AV Sinha S Kaye L . Global burden of chronic obstructive pulmonary disease through 2050. JAMA Netw Open. (2023) 6:e2346598. doi: 10.1001/jamanetworkopen.2023.46598, PMID: 38060225 PMC10704283

[13] De Oca MM Perez-Padilla R Celli B Aaron SD Wehrmeister FC Amaral AFS . The global burden of COPD: epidemiology and effect of prevention strategies. Lancet Respir Med. (2025) 13:709–24. doi: 10.1016/S2213-2600(24)00339-4, PMID: 40684784

[14] Decramer M Janssens W Miravitlles M . Chronic obstructive pulmonary disease. Lancet. (2012) 379:1341–51. doi: 10.1016/S0140-6736(11)60968-9, PMID: 22314182 PMC7172377

[15] Kim V Criner GJ . Chronic bronchitis and chronic obstructive pulmonary disease. Am J Respir Crit Care Med. (2013) 187:228–37. doi: 10.1164/rccm.201210-1843CI, PMID: 23204254 PMC4951627

[16] Martinon F Burns K Tschopp J . The inflammasome. Mol Cell. (2002) 10:417–26. doi: 10.1016/S1097-2765(02)00599-3, PMID: 12191486

[17] Bateman G Hill B Knight R Boucher D . Great balls of fire: activation and signalling of inflammatory caspases. Biochem Soc Trans. (2021) 49:1311–24. doi: 10.1042/BST20200986, PMID: 34060593 PMC8286819

[18] Fu J Wu H . Structural mechanisms of NLRP3 inflammasome assembly and activation. Annu Rev Immunol. (2023) 41:301–16. doi: 10.1146/annurev-immunol-081022-021207, PMID: 36750315 PMC10159982

[19] Swanson KV Deng M Ting JPY . The NLRP3 inflammasome: molecular activation and regulation to therapeutics. Nat Rev Immunol. (2019) 19:477–89. doi: 10.1038/s41577-019-0165-0, PMID: 31036962 PMC7807242

[20] McKee CM Coll RC . NLRP3 inflammasome priming: A riddle wrapped in a mystery inside an enigma. J Leukoc Biol. (2020) 108:937–52. doi: 10.1002/JLB.3MR0720-513R, PMID: 32745339

[21] Ji J Hou J Xia Y Xiang Z Han X . NLRP3 inflammasome activation in alveolar epithelial cells promotes myofibroblast differentiation of lung-resident mesenchymal stem cells during pulmonary fibrogenesis. Biochim Biophys Acta BBA - Mol Basis Dis. (2021) 1867:166077. doi: 10.1016/j.bbadis.2021.166077, PMID: 33515677

[22] Vithalkar MP Pradhan S Sandra KS Bharath HB Nayak Y . Modulating NLRP3 inflammasomes in idiopathic pulmonary fibrosis: A comprehensive review on flavonoid-based interventions. Cell Biochem Biophys. (2025) 83:2669–701. doi: 10.1007/s12013-025-01696-4, PMID: 39966334 PMC12414096

[23] Gu L Zhu J Nie Q Xie B Xue S Zhang A . NLRP3 promotes inflammatory signaling and IL-1β cleavage in acute lung injury caused by cell wall extract of Lactobacillus casei. Commun Biol. (2025) 8:20. doi: 10.1038/s42003-025-07462-9, PMID: 39774843 PMC11706994

[24] Ito H Kimura H Karasawa T Hisata S Sadatomo A Inoue Y . NLRP3 inflammasome activation in lung vascular endothelial cells contributes to intestinal ischemia/reperfusion-induced acute lung injury. J Immunol. (2020) 205:1393–405. doi: 10.4049/jimmunol.2000217, PMID: 32727891

[25] Ciminieri C Woest ME Reynaert NL Heijink IH Wardenaar R Spierings DCJ . IL-1β Induces a proinflammatory fibroblast microenvironment that impairs lung progenitors’ Function. Am J Respir Cell Mol Biol. (2023) 68:444–55. doi: 10.1165/rcmb.2022-0209OC, PMID: 36608844 PMC12042164

[26] Churg A Zhou S Wang X Wang R Wright JL . The role of interleukin-1β in murine cigarette smoke–induced emphysema and small airway remodeling. Am J Respir Cell Mol Biol. (2009) 40:482–90. doi: 10.1165/rcmb.2008-0038OC, PMID: 18931327

[27] Faner R Sobradillo P Noguera A Gomez C Cruz T López-Giraldo A . The inflammasome pathway in stable COPD and acute exacerbations. ERJ Open Res. (2016) 2:00002–2016. doi: 10.1183/23120541.00002-2016, PMID: 27730204 PMC5034597

[28] Eltom S Stevenson CS Rastrick J Dale N Raemdonck K Wong S . P2X7 receptor and caspase 1 activation are central to airway inflammation observed after exposure to tobacco smoke. PloS One. (2011) 6:e24097. doi: 10.1371/journal.pone.0024097, PMID: 21915284 PMC3167831

[29] Moon HG Cao Y Yang J Lee JH Choi HS Jin Y . Lung epithelial cell-derived extracellular vesicles activate macrophage-mediated inflammatory responses via ROCK1 pathway. Cell Death Dis. (2015) 6:e2016–6. doi: 10.1038/cddis.2015.282, PMID: 26658190 PMC4720875

[30] Lee H Groot M Pinilla-Vera M Fredenburgh LE Jin Y . Identification of miRNA-rich vesicles in bronchoalveolar lavage fluid: Insights into the function and heterogeneity of extracellular vesicles. J Controlled Release. (2019) 294:43–52. doi: 10.1016/j.jconrel.2018.12.008, PMID: 30529727 PMC6372374

[31] Nicod LP . Lung defences: an overview. Eur Respir Rev. (2005) 14:45–50. doi: 10.1183/09059180.05.00009501

[32] Toews GB . Impact of bacterial infections on airway diseases. Eur Respir Rev. (2005) 14:62–8. doi: 10.1183/09059180.05.00009504

[33] Hill AT Campbell EJ Hill SL Bayley DL Stockley RA . Association between airway bacterial load and markers of airway inflammation in patients with stable chronic bronchitis. Am J Med. (2000) 109:288–95. doi: 10.1016/S0002-9343(00)00507-6, PMID: 10996579

[34] Barker BL Haldar K Patel H Pavord ID Barer MR Brightling CE . Association between pathogens detected using quantitative polymerase chain reaction with airway inflammation in COPD at stable state and exacerbations. Chest. (2015) 147:46–55. doi: 10.1378/chest.14-0764, PMID: 25103335 PMC4285081

[35] Wilkinson TMA Aris E Bourne S Clarke SC Peeters M Pascal TG . A prospective, observational cohort study of the seasonal dynamics of airway pathogens in the aetiology of exacerbations in COPD. Thorax. (2017) 72:919–27. doi: 10.1136/thoraxjnl-2016-209023, PMID: 28432209 PMC5738531

[36] Garcha DS Thurston SJ Patel ARC Mackay AJ Goldring JJP Donaldson GC . Changes in prevalence and load of airway bacteria using quantitative PCR in stable and exacerbated COPD. Thorax. (2012) 67:1075–80. doi: 10.1136/thoraxjnl-2012-201924, PMID: 22863758

[37] Erwin AL Smith AL . Nontypeable Haemophilus influenzae: understanding virulence and commensal behavior. Trends Microbiol. (2007) 15:355–62. doi: 10.1016/j.tim.2007.06.004, PMID: 17600718

[38] Berenson CS Wrona CT Grove LJ Maloney J Garlipp MA Wallace PK . Impaired alveolar macrophage response to haemophilus antigens in chronic obstructive lung disease. Am J Respir Crit Care Med. (2006) 174:31–40. doi: 10.1164/rccm.200509-1461OC, PMID: 16574934 PMC2662920

[39] Essilfie AT Simpson JL Dunkley ML Morgan LC Oliver BG Gibson PG . Combined *Haemophilus influenzae* respiratory infection and allergic airways disease drives chronic infection and features of neutrophilic asthma. Thorax. (2012) 67:588–99. doi: 10.1136/thoraxjnl-2011-200160, PMID: 22387445

[40] Berenson CS Murphy TF Wrona CT Sethi S . Outer membrane protein P6 of nontypeable *haemophilus influenzae* is a potent and selective inducer of human macrophage proinflammatory cytokines. Infect Immun. (2005) 73:2728–35. doi: 10.1128/IAI.73.5.2728-2735.2005, PMID: 15845475 PMC1087348

[41] Taylor AE Finney-Hayward TK Quint JK Thomas CMR Tudhope SJ Wedzicha JA . Defective macrophage phagocytosis of bacteria in COPD. Eur Respir J. (2010) 35:1039–47. doi: 10.1183/09031936.00036709, PMID: 19897561

[42] Rotta Detto Loria J Rohmann K Droemann D Kujath P Rupp J Goldmann T . Nontypeable haemophilus influenzae infection upregulates the NLRP3 inflammasome and leads to caspase-1-dependent secretion of interleukin-1β — A possible pathway of exacerbations in COPD. PloS One. (2013) 8:e66818. doi: 10.1371/journal.pone.0066818, PMID: 23840534 PMC3694113

[43] Lane S White TLA Walsh EE Cattley RT Cumberland R Hawse WF . Antiviral epithelial-macrophage crosstalk permits secondary bacterial infections. mBio. (2023) 14:e00863–23. doi: 10.1128/mbio.00863-23, PMID: 37772820 PMC10653878

[44] Linden DA Guo-Parke H McKelvey MC Einarsson GG Lee AJ Fairley DJ . Valaciclovir for epstein-barr virus suppression in moderate-to-severe COPD. CHEST. (2023) 164:625–36. doi: 10.1016/j.chest.2023.03.040, PMID: 37011709 PMC10808072

[45] Fuss IJ Kanof ME Smith PD Zola H . Isolation of whole mononuclear cells from peripheral blood and cord blood. Curr Protoc Immunol. (2009) 85:711–8. doi: 10.1002/0471142735.im0701s85, PMID: 19347849

[46] Eitan E Zhang S Witwer KW Mattson MP . Extracellular vesicle–depleted fetal bovine and human sera have reduced capacity to support cell growth. J Extracell Vesicles. (2015) 4:26373. doi: 10.3402/jev.v4.26373, PMID: 25819213 PMC4376846

[47] R Core Team . R: A Language and Environment for Statistical Computing (2022). Vienna, Austria: R Foundation for Statistical Computing. Available online at: https://www.R-project.org (Accessed October 24, 2024).

[48] Zhang X Smits AH Van Tilburg GB Ovaa H Huber W Vermeulen M . Proteome-wide identification of ubiquitin interactions using UbIA-MS. Nat Protoc. (2018) 13:530–50. doi: 10.1038/nprot.2017.147, PMID: 29446774

[49] Chen SY Feng Z Yi X . A general introduction to adjustment for multiple comparisons. J Thorac Dis. (2017) 9:1725–9. doi: 10.21037/jtd.2017.05.34, PMID: 28740688 PMC5506159

[50] Xie Z Bailey A Kuleshov MV Clarke DJB Evangelista JE Jenkins SL . Gene set knowledge discovery with enrichr. Curr Protoc. (2021) 1:e90. doi: 10.1002/cpz1.90, PMID: 33780170 PMC8152575

[51] Kuleshov MV Jones MR Rouillard AD Fernandez NF Duan Q Wang Z . Enrichr: a comprehensive gene set enrichment analysis web server 2016 update. Nucleic Acids Res. (2016) 44:W90–7. doi: 10.1093/nar/gkw377, PMID: 27141961 PMC4987924

[52] Chen EY Tan CM Kou Y Duan Q Wang Z Meirelles GV . Enrichr: interactive and collaborative HTML5 gene list enrichment analysis tool. BMC Bioinf. (2013) 14:128. doi: 10.1186/1471-2105-14-128, PMID: 23586463 PMC3637064

[53] Sellin Jeffries MK Kiss AJ Smith AW Oris JT . A comparison of commercially-available automated and manual extraction kits for the isolation of total RNA from small tissue samples. BMC Biotechnol. (2014) 14:94. doi: 10.1186/s12896-014-0094-8, PMID: 25394494 PMC4239376

[54] Love MI Huber W Anders S . Moderated estimation of fold change and dispersion for RNA-seq data with DESeq2. Genome Biol. (2014) 15:550. doi: 10.1186/s13059-014-0550-8, PMID: 25516281 PMC4302049

[55] Zhu A Ibrahim JG Love MI . Heavy-tailed prior distributions for sequence count data: removing the noise and preserving large differences. Bioinformatics. (2019) 35:2084–92. doi: 10.1093/bioinformatics/bty895, PMID: 30395178 PMC6581436

[56] Oliveros JC Venny . An interactive tool for comparing lists with Venn’s diagrams (2007). Available online at: https://bioinfogp.cnb.csic.es/tools/venny/index.html (Accessed January 29, 2025).

[57] Blighe K . EnhancedVolcano (2018). Available online at: https://bioconductor.org/packages/EnhancedVolcano (Accessed October 24, 2024).

[58] Diamant I Clarke DJB Evangelista JE Lingam N Ma’ayan A . Harmonizome 3.0: integrated knowledge about genes and proteins from diverse multi-omics resources. Nucleic Acids Res. (2024) 53:D1016–D1028. doi: 10.1093/nar/gkae1080, PMID: 39565209 PMC11701526

[59] Rouillard AD Gundersen GW Fernandez NF Wang Z Monteiro CD McDermott MG . The harmonizome: a collection of processed datasets gathered to serve and mine knowledge about genes and proteins. Database. (2016) 2016:baw100. doi: 10.1093/database/baw100, PMID: 27374120 PMC4930834

[60] Campbell JD Luo L Liu G Xiao J Gerrein J Guardela BJ . Characterizing the small RNA transcriptome associated with COPD and ILD using next-generation sequencing. BMC Proc. (2012) 6. doi: 10.1186/1753-6561-6-S6-P6

[61] Sundar IK Li D Rahman I . Small RNA-sequence analysis of plasma-derived extracellular vesicle miRNAs in smokers and patients with chronic obstructive pulmonary disease as circulating biomarkers. J Extracell Vesicles. (2019) 8:1684816. doi: 10.1080/20013078.2019.1684816, PMID: 31762962 PMC6848892

[62] Osei ET Florez-Sampedro L Timens W Postma DS Heijink IH Brandsma CA . Unravelling the complexity of COPD by microRNAs: it’s a small world after all. Eur Respir J. (2015) 46:807–18. doi: 10.1183/13993003.02139-2014, PMID: 26250493

[63] Devulder J Baker JR Monkley S Odqvist L Donnelly LE Barnes PJ . Extracellular vesicles produced by airway epithelial cells from COPD patients contain miRNAs involved in cellular senescence. ERJ Open Res. (2022) 8:217. doi: 10.1183/23120541.LSC-2022.217

[64] Gou Z Yang H Wang R Wang S Chen Q Liu Z . A new frontier in precision medicine: Exploring the role of extracellular vesicles in chronic obstructive pulmonary disease. BioMed Pharmacother. (2024) 174:116443. doi: 10.1016/j.biopha.2024.116443, PMID: 38513597

[65] Xie T Liang J Stripp B Noble PW . Cell–cell interactions and communication dynamics in lung fibrosis. Chin Med J Pulm Crit Care Med. (2024) 2:63–71. doi: 10.1016/j.pccm.2024.04.001, PMID: 39169931 PMC11332853

[66] Wang L Yu Q Xiao J Chen Q Fang M Zhao H . Cigarette Smoke Extract-Treated Mouse Airway Epithelial Cells-Derived Exosomal LncRNA MEG3 Promotes M1 Macrophage Polarization and Pyroptosis in Chronic Obstructive Pulmonary Disease by Upregulating TREM-1 via m^6^ A Methylation. Immune Netw. (2024) 24:e3. doi: 10.4110/in.2024.24.e3, PMID: 38725674 PMC11076299

[67] Dubey SR Turnbull C Pandey A Zhao A Kurera M Al-Zidan R . Molecular mechanisms and regulation of inflammasome activation and signaling: sensing of pathogens and damage molecular patterns. Cell Mol Immunol. (2025) 22:1313–44. doi: 10.1038/s41423-025-01354-y, PMID: 41062723 PMC12575685

[68] Wang X Eagen WJ Lee JC . Orchestration of human macrophage NLRP3 inflammasome activation by Staphylococcus aureus extracellular vesicles. Proc Natl Acad Sci. (2020) 117:3174–84. doi: 10.1073/pnas.1915829117, PMID: 31988111 PMC7022218

[69] Forrest OA Dobosh B Ingersoll SA Rao S Rojas A Laval J . Neutrophil-derived extracellular vesicles promote feed-forward inflammasome signaling in cystic fibrosis airways. J Leukoc Biol. (2022) 112:707–16. doi: 10.1002/JLB.3AB0321-149R, PMID: 35172381

[70] Useckaite Z Ward MP Trappe A Reilly R Lennon J Davage H . Increased extracellular vesicles mediate inflammatory signalling in cystic fibrosis. Thorax. (2020) 75:449–58. doi: 10.1136/thoraxjnl-2019-214027, PMID: 32265339 PMC7279202

[71] Stack JH Beaumont K Larsen PD Straley KS Henkel GW Randle JCR . IL-converting enzyme/caspase-1 inhibitor VX-765 blocks the hypersensitive response to an inflammatory stimulus in monocytes from familial cold autoinflammatory syndrome patients. J Immunol. (2005) 175:2630–4. doi: 10.4049/jimmunol.175.4.2630, PMID: 16081838

[72] Wannamaker W Davies R Namchuk M Pollard J Ford P Ku G . (S)-1-((S)-2-{[1-(4-amino-3-chloro-phenyl)-methanoyl]-amino}-3,3-dimethyl-butanoyl)-pyrrolidine-2-carboxylic acid ((2 R,3 S)-2-ethoxy-5-oxo-tetrahydro-furan-3-yl)-amide (VX-765), an orally available selective interleukin (IL)-converting enzyme/caspase-1 inhibitor, exhibits potent anti-inflammatory activities by inhibiting the release of IL-1β and IL-18. J Pharmacol Exp Ther. (2007) 321:509–16. doi: 10.1124/jpet.106.111344, PMID: 17289835

[73] Coll RC Robertson AAB Chae JJ Higgins SC Muñoz-Planillo R Inserra MC . A small-molecule inhibitor of the NLRP3 inflammasome for the treatment of inflammatory diseases. Nat Med. (2015) 21:248–55. doi: 10.1038/nm.3806, PMID: 25686105 PMC4392179

[74] Coll RC Hill JR Day CJ Zamoshnikova A Boucher D Massey NL . MCC950 directly targets the NLRP3 ATP-hydrolysis motif for inflammasome inhibition. Nat Chem Biol. (2019) 15:556–9. doi: 10.1038/s41589-019-0277-7, PMID: 31086327

[75] McKenzie BA Mamik MK Saito LB Boghozian R Monaco MC Major EO . Caspase-1 inhibition prevents glial inflammasome activation and pyroptosis in models of multiple sclerosis. Proc Natl Acad Sci. (2018) 115:e6065–74. doi: 10.1073/pnas.1722041115, PMID: 29895691 PMC6042136

[76] Jiao M Wang J Liu W Zhao X Qin Y Zhang C . VX-765 inhibits pyroptosis and reduces inflammation to prevent acute liver failure by upregulating PPARα expression. Ann Hepatol. (2023) 28:101082. doi: 10.1016/j.aohep.2023.101082, PMID: 36893888

[77] Alghamdi M Alamry SA Bahlas SM Uversky VN Redwan EM . Circulating extracellular vesicles and rheumatoid arthritis: a proteomic analysis. Cell Mol Life Sci. (2022) 79:25. doi: 10.1007/s00018-021-04020-4, PMID: 34971426 PMC11072894

[78] Wang S Kojima K Mobley JA West AB . Proteomic analysis of urinary extracellular vesicles reveal biomarkers for neurologic disease. EBioMedicine. (2019) 45:351–61. doi: 10.1016/j.ebiom.2019.06.021, PMID: 31229437 PMC6642358

[79] BarraChina MN Calderón-Cruz B Fernandez-Rocca L García Á . Application of extracellular vesicles proteomics to cardiovascular disease: guidelines, data analysis, and future perspectives. PROTEOMICS. (2019) 19:1800247. doi: 10.1002/pmic.201800247, PMID: 30467982

[80] Wedzicha JA Seemungal TA . COPD exacerbations: defining their cause and prevention. Lancet. (2007) 370:786–96. doi: 10.1016/S0140-6736(07)61382-8, PMID: 17765528 PMC7134993

[81] Murphy TF . Vaccines for nontypeable haemophilus influenzae: the future is now. Papasian CJ editor Clin Vaccine Immunol. (2015) 22:459–66. doi: 10.1128/CVI.00089-15, PMID: 25787137 PMC4412935

[82] Ashburner M Ball CA Blake JA Botstein D Butler H Cherry JM . Gene Ontology: tool for the unification of biology. Nat Genet. (2000) 25:25–9. doi: 10.1038/75556, PMID: 10802651 PMC3037419

[83] The Gene Ontology Consortium Aleksander SA Balhoff J Carbon S Cherry JM Drabkin HJ . The Gene Ontology knowledgebase in 2023. Baryshnikova A, editor. GENETICS. (2023) 224:iyad031. doi: 10.1093/genetics/iyad031, PMID: 36866529 PMC10158837

[84] Ridyard KE Overhage J . The potential of human peptide LL-37 as an antimicrobial and anti-biofilm agent. Antibiotics. (2021) 10:650. doi: 10.3390/antibiotics10060650, PMID: 34072318 PMC8227053

[85] Roby KD Di Nardo A . Innate immunity and the role of the antimicrobial peptide cathelicidin in inflammatory skin disease. Drug Discov Today Dis Mech. (2013) 10:e79–82. doi: 10.1016/j.ddmec.2013.01.001, PMID: 24489580 PMC3904447

[86] Xu D Lu W . Defensins: A double-edged sword in host immunity. Front Immunol. (2020) 11:764. doi: 10.3389/fimmu.2020.00764, PMID: 32457744 PMC7224315

[87] Lee J Lu Y Mohammad N Han K Brantly ML . The exacerbation of the inflammatory response to nontypeable haemophilus influenzae bacteria by alpha-defensins. Am J Respir Crit Care Med;. (2024) 209:A4297. doi: 10.1164/ajrccm-conference.2024.B70

[88] Chen X Deng S Wang W Castiglione S Duan Z Luo L . Human antimicrobial peptide LL-37 contributes to Alzheimer’s disease progression. Mol Psychiatry. (2022) 27:4790–9. doi: 10.1038/s41380-022-01790-6, PMID: 36138130

[89] Neshani A Zare H Akbari Eidgahi MR Kamali Kakhki R Safdari H Khaledi A . LL-37: Review of antimicrobial profile against sensitive and antibiotic-resistant human bacterial pathogens. Gene Rep. (2019) 17:100519. doi: 10.1016/j.genrep.2019.100519

[90] Scott MG Davidson DJ Gold MR Bowdish D Hancock REW . The human antimicrobial peptide LL-37 is a multifunctional modulator of innate immune responses. J Immunol. (2002) 169:3883–91. doi: 10.4049/jimmunol.169.7.3883, PMID: 12244186

[91] Brown KL Poon GFT Birkenhead D Pena OM Falsafi R Dahlgren C . Host defense peptide LL-37 selectively reduces proinflammatory macrophage responses. J Immunol. (2011) 186:5497–505. doi: 10.4049/jimmunol.1002508, PMID: 21441450

[92] Valenti P Antonini G . Lactoferrin: Lactoferrin: an important host defence against microbial and viral attack. Cell Mol Life Sci. (2005) 62:2576–87. doi: 10.1007/s00018-005-5372-0, PMID: 16261253 PMC11139069

[93] Kaczyńska K Jampolska M Wojciechowski P Sulejczak D Andrzejewski K Zając D . Potential of lactoferrin in the treatment of lung diseases. Pharmaceuticals. (2023) 16:192. doi: 10.3390/ph16020192, PMID: 37259341 PMC9960651

[94] Bukowska-Ośko I Sulejczak D Kaczyńska K Kleczkowska P Kramkowski K Popiel M . Lactoferrin as a human genome “Guardian”—An overall point of view. Int J Mol Sci. (2022) 23:5248., PMID: 35563638 10.3390/ijms23095248 PMC9105968

[95] Kowalczyk P Kaczyńska K Kleczkowska P Bukowska-Ośko I Kramkowski K Sulejczak D . The lactoferrin phenomenon—A miracle molecule. Molecules. (2022) 27:2941. doi: 10.3390/molecules27092941, PMID: 35566292 PMC9104648

[96] Vargas Buonfiglio LG Borcherding JA Frommelt M Parker GJ Duchman B Vanegas Calderón OG . Airway surface liquid from smokers promotes bacterial growth and biofilm formation via iron-lactoferrin imbalance. Respir Res. (2018) 19:42. doi: 10.1186/s12931-018-0743-x, PMID: 29524964 PMC5845328

[97] Donoghue LJ Markovetz MR Morrison CB Chen G McFadden KM Sadritabrizi T . BPIFB1 loss alters airway mucus properties and diminishes mucociliary clearance. Am J Physiol-Lung Cell Mol Physiol. (2023) 325:L765–75. doi: 10.1152/ajplung.00390.2022, PMID: 37847709 PMC11068428

[98] Chen M Liu L . High expression of bpifb1 stimulates macrophages to secrete inflammatory factors and inducing pulmonary fibrosis. Eur Respir Soc. (2024) 64:PA2638. doi: 10.1183/13993003.congress-2024.PA2638

[99] Titz B Sewer A Schneider T Elamin A Martin F Dijon S . Alterations in the sputum proteome and transcriptome in smokers and early-stage COPD subjects. J Proteomics. (2015) 128:306–20. doi: 10.1016/j.jprot.2015.08.009, PMID: 26306861

[100] Ghafouri B Ståhlbom B Tagesson C Lindahl M . Newly identified proteins in human nasal lavage fluid from non-smokers and smokers using two-dimensional gel electrophoresis and peptide mass fingerprinting. Proteomics. (2002) 2:112–20. doi: 10.1002/1615-9861(200201)2:1<112::AID-PROT112>3.0.CO;2-N, PMID: 11788998

[101] Yang S Yin Y Xu W Zhang X Gao Y Liao H . Type I interferon induced by DNA of nontypeable Haemophilus influenza modulates inflammatory cytokine profile to promote susceptibility to this bacterium. Int Immunopharmacol. (2019) 74:105710. doi: 10.1016/j.intimp.2019.105710, PMID: 31255879

[102] Yang H Wen X Wu F Zheng Y Dai C Zhao N . Inter-relationships among neutrophilic inflammation, air trapping and future exacerbation in COPD: an analysis of ECOPD study. BMJ Open Respir Res. (2023) 10:e001597. doi: 10.1136/bmjresp-2022-001597, PMID: 37028910 PMC10083880

[103] Metzemaekers M Gouwy M Proost P . Neutrophil chemoattractant receptors in health and disease: double-edged swords. Cell Mol Immunol. (2020) 17:433–50. doi: 10.1038/s41423-020-0412-0, PMID: 32238918 PMC7192912

[104] Huang H Huang X Zeng K Deng F Lin C Huang W . Interleukin-6 is a strong predictor of the frequency of COPD exacerbation within 1 year. Int J Chron Obstruct Pulmon Dis. (2021) 16:2945–51. doi: 10.2147/COPD.S332505, PMID: 34737559 PMC8560075

[105] Ono Y Fujino N Saito T Matsumoto S Konno S Endo T . Characterization of IL-6R-expressing monocytes in the lung of patients with chronic obstructive pulmonary disease. Respir Investig. (2024) 62:856–66. doi: 10.1016/j.resinv.2024.07.013, PMID: 39068895

[106] Johnston S Molyneaux P Singanayagam A Beasley J Mallia P . Lung microbiology and exacerbations in COPD. Int J Chron Obstruct Pulmon Dis. (2012) 555:555–69. doi: 10.2147/COPD.S28286, PMID: 22969296 PMC3437812

[107] Theodorakis N Feretzakis G Hitas C Kreouzi M Kalantzi S Spyridaki A . Immunosenescence: how aging increases susceptibility to bacterial infections and virulence factors. Microorganisms. (2024) 12:2052. doi: 10.3390/microorganisms12102052, PMID: 39458361 PMC11510421

[108] Amand M Adams P Schober R Iserentant G Servais JY Moutschen M . The anti-caspase 1 inhibitor VX-765 reduces immune activation, CD4+ T cell depletion, viral load, and total HIV-1 DNA in HIV-1 infected humanized mice. eLife. (2023) 12:e83207. doi: 10.7554/eLife.83207, PMID: 36800238 PMC9937651

[109] Jin Y Liu Y Xu L Xu J Xiong Y Peng Y . Novel role for caspase 1 inhibitor VX765 in suppressing NLRP3 inflammasome assembly and atherosclerosis via promoting mitophagy and efferocytosis. Cell Death Dis. (2022) 13:512. doi: 10.1038/s41419-022-04966-8, PMID: 35641492 PMC9156694

[110] Sun Z Gu L Wu K Wang K Ru J Yang S . VX-765 enhances autophagy of human umbilical cord mesenchymal stem cells against stroke-induced apoptosis and inflammatory responses via AMPK/mTOR signaling pathway. CNS Neurosci Ther. (2020) 26:952–61. doi: 10.1111/cns.13400, PMID: 32459063 PMC7415204

[111] Wen S Deng F Li L Xu L Li X Fan Q . VX-765 ameliorates renal injury and fibrosis in diabetes by regulating caspase-1-mediated pyroptosis and inflammation. J Diabetes Investig. (2022) 13:22–33. doi: 10.1111/jdi.13660, PMID: 34494385 PMC8756311

[112] MacKenzie SH Schipper JL Clark AC . The potential for caspases in drug discovery. Curr Opin Drug Discov Devel. (2010) 13:568–76. PMC3289102 20812148

[113] Mangan MSJ Olhava EJ Roush WR Seidel HM Glick GD Latz E . Targeting the NLRP3 inflammasome in inflammatory diseases. Nat Rev Drug Discov. (2018) 17:588–606. doi: 10.1038/nrd.2018.97, PMID: 30026524

[114] Liu Q Li D Pan X Liang Y . Targeted therapy using engineered extracellular vesicles: principles and strategies for membrane modification. J Nanobiotechnology. (2023) 21:334. doi: 10.1186/s12951-023-02081-0, PMID: 37717008 PMC10505332

[115] Su X Wang H Li Q Chen Z . Extracellular vesicles: A review of their therapeutic potentials, sources, biodistribution, and administration routes. Int J Nanomedicine. (2025) 20:3175–99. doi: 10.2147/IJN.S502591, PMID: 40098717 PMC11913029

[116] Kumar MA Baba SK Sadida HQ Marzooqi SAL Jerobin J Altemani FH . Extracellular vesicles as tools and targets in therapy for diseases. Signal Transduct Target Ther. (2024) 9:27. doi: 10.1038/s41392-024-01735-1, PMID: 38311623 PMC10838959

