# Supplementary material for: Bronchial epithelial cell-derived extracellular vesicles drive inflammasome activation and NTHi infection in COPD
Source: Front Immunol. 2026 Jan 2;16:1713012. doi: 10.3389/fimmu.2025.1713012 (PMC12807989; doi:10.3389/fimmu.2025.1713012)
Supplement: Supplementary file 1 [file DataSheet1.docx]

Supplementary Material

# Supplementary 1: COPD donor sample information

| Donor | Sex | Age (years) | FEV_1_ % predicted | FEV_1_/FVC | TLCO | GOLD | Current smoker |
| --- | --- | --- | --- | --- | --- | --- | --- |
| 01 | Male | 39 | 80 | 62.58 | 96 | 1 | No |
| 02 | Male | 56 | 76 | 67.99 | 53 | 2 | Yes |
| 03 | Male | 57 | 75 | 64.79 | 67 | 3 | Yes |
| 04 | Female | 61 | 68 | 58.85 | 50 | 2 | No |
| 05 | Male | 64 | 62 | 53.73 | 81 | 2 | No |
| 06 | Male | 64 | 39 | 44 | NA | 4 | No |
| 07 | Male | 67 | 56 | 44.52 | 58 | 2 | Yes |

Unavailable data marked by NA – not available, this patient was unable to record technically reproducible measurements at the time of sample collection. Abbreviations: FEV_1_ – forced expiratory volume in one second, FVC - Forced Vital Capacity, TLCO - Transfer Factor for Carbon Monoxide, GOLD - Global Initiative for Chronic Obstructive Lung Disease.

# Supplementary 2: Healthy donor sample information

| Donor | Sex | Age | Current smoker |
| --- | --- | --- | --- |
| 08 | Female | 54 | No |
| 09 | Male | 63 | No |
| 10 | Male | 69 | No |
| 11 | Male | 79 | No |
| 12 | Male | 85 | No |

# Supplementary 3:

## TEM staining

Formvar/carbon-coated copper grids (Agar Scientific) were incubated with 10 μL of EVs in PBS for 5 mins on clean parafilm. The EV solution was carefully blotted to remove excess liquid. The grid was touched to a drop of Uranyless (Electron Microscopy Sciences, Cat#22409), immediately blotted, then again touched to a drop of Uranyless, this time for 20s before blotting. The grids with EVs were then left to dry for 1hr minimum before imaging using the TEM (TEM-JEM-1400Plus).

## ELISA

Plates were read at 450nm, with a wavelength correction at 570 nm. The wavelength corrected values were calculated (450 nm values − 570n m values), and sample concentrations calculated with reference to the standard curve values. ELISA kits used are shown below:

| Target | Standard range | Manufacturer |
| --- | --- | --- |
| IL-8 | 31.25 – 2000pg/mL | R&D |
| TNF-α | 7.813 – 500pg/mL | Invitrogen |
| IL-1β | 2.344 – 150pg/mL | Invitrogen |
| IL-18 | 5.859 – 750pg/mL | R&D |

## NTHi culture

Brain heart infusion (BHI) broth was prepared by the addition of 37g of BHI (ThermoFisher Scientific, #CM1135B) to 1L distilled water and autoclaved. Chocolate agar was prepared by adding 40g of Blood Agar Base (ThermoFisher Scientific, #CM0055B) into 1L distilled water and autoclaved. Once autoclaved and while still hot, 10% sheep blood was added and the agar mix and placed in a water bath at 80°C. Once at 80°C, the mixture was poured into sterile petri dishes and allowed to set under a Bunsen burner.

## LDH

Plates were read at 490 nm and 680 nm. The values were calculated to correct for background signal (490 nm values – 680 nm values), and LDH release calculated with reference to maximum lysis controls.

# Supplementary 4: Inflammatory cytokines in *NTHi* infected THP-1 cells

*** Supplementary 4: Inflammatory cytokine release in response to NTHi infection*** *THP-1 cells were infected with NTHi at MOI10. The levels of TNF-α (****A****), IL-8 (****B****), IL-1β* ***(C****), and IL-6 (D) release were quantified by ELISA. Data were analysed by unpaired t test.* *Statistical significance was indicated as follows: * P < 0.05, ** P < 0.01, *** P < 0.001. Any P values > 0.05 were indicated using ns (not significant).*
